# Spatiotemporal structure and composition of the microbial communities in hypersaline Lake Magadi, Kenya

**DOI:** 10.12688/f1000research.134465.2

**Published:** 2024-05-17

**Authors:** Alex Kipnyargis, Eucharia Kenya, Fathiya Khamis, Romano Mwirichia

**Affiliations:** 1 Department of Biological Sciences, University of Embu, Embu, Kenya; 2 Arthropod Pathology, International Centre of Insect Physiology and Ecology, Nairobi, Nairobi County, Kenya

**Keywords:** Soda lake, spatiotemporal, archaea, bacteria, physicochemical parameters, α-diversity, β-diversity

## Abstract

**Background:**

Soda lakes are habitats characterized by haloalkaline conditions also known to host unique microbial communities. The water chemistry changes with seasons due to evaporative concentration or floods from the surrounding grounds. However, it is not yet clear if the change in physiochemical changes influences the spatiotemporal diversity and structure of microbial communities in these ecosystems.

**Methods:**

Using 16S rRNA gene amplicon sequencing, we investigated the diversity and structure of microbial communities in water and brine samples taken from Lake Magadi between June and September 2018. Additionally, physicochemical parameters were also analyzed for every sampling site. Additionally, physicochemical parameters were also analyzed for every sampling site.

**Results:**

The abundant bacterial phyla were Proteobacteria, Cyanobacteria, Bacteroidetes, Actinobacteria, Firmicutes, Verrumicrobia, Deinococcus-Thermus, Spirochaetes, and Chloroflexi. The Archaeal diversity was represented by phyla Euryachaeota, Crenarchaeota, Euryarchaeota, and Thaumarchaeota. The dominant bacterial species were: *Euhalothece* sp. (10.3%), *Rhodobaca* sp. (9.6%), *Idiomarina* sp. (5.8%), *Rhodothermus* sp. (3.0%), *Roseinatronobacter* sp. (2.4%), *Nocardioides* sp. (2.3%), *Gracilimonas* sp. (2.2%), and *Halomonas* sp. (2%). The dominant archaeal species included *Halorubrum* sp. (18.3%), *Salinarchaeum* sp. (5.3%), and *Haloterrigena* sp. (1.3%). The composition of bacteria was higher than that of archaea, while their richness and diversity varied widely across the sampling seasons. The α-diversity indices showed that high diversity was recorded in August, followed by September, June, and July in that order. The findings demonstrated that temperature, pH, P+, K+, NO3 -, and total dissolved solids (TDS) contributed majorly to the diversity observed in the microbial community. Multivariate analysis revealed significant spatial and temporal effects on β-diversity and salinity and alkalinity were the major drivers of microbial composition in Lake Magadi.

**Conclusions:**

We provide insights into the relationships between microbial structure and geochemistry across various sampling sites in Lake Magadi.

## Introduction

Most living organisms are adapted to habitats characterized by moderate temperature (10–37°C), pH (of approximately 7), salinity (0.15–0.5 M NaCl), pressure (1 atm), and adequate supply of water ( Aguilar *et al.*, 1998; Antranikian *et al.*, 2005). However, molecular techniques such as next-generation sequencing have revealed that diverse groups of organisms thrive even in biomes previously thought to be lifeless ( Canganella & Wiegel, 2011; Rampelotto, 2013). Microbial communities in ecosystems such as the hypersaline lakes of the East African Rift Valley survive and thrive under one or several extreme conditions and are referred to as polyextremophiles ( Sorokin *et al.*, 2014a; Urbieta *et al.*, 2015).

The distribution and diversity of microbial communities in hypersaline lakes are mainly affected by physicochemical parameters ( Tazi *et al.*, 2014). Lake Magadi is an example of an extreme habitat characterized by high concentrations of Na ^+^, K ^+^, CO _3_^2–^, Cl ^–^, HCO _3_^–^, and SiO _2,_ but low concentrations of Ca ^2+^ and Mg ^2+^ ( Jones *et al.*, 1998; Getenet *et al.*, 2022). During the dry seasons, thermonatrite (Na _2_CO _3_.H _2_O), and halite (NaCl) precipitate by evaporative concentration ( Eugester, 1971, 1980). The lake is in a region with alternating wet and dry seasons. During the dry season, when ground temperatures exceed 40°С, there is extensive evaporation ( Matagi, 2004; Muruga & Anyango, 2013). In addition, a white layer of soda covers the lake almost completely, and rainy days may result in flooding from the surrounding water entering the lake.

Despite the extreme conditions existing in the lake, it is a highly productive ecosystem with diverse microbial communities driving active nitrogen, carbon, and sulfur cycles ( Jones *et al.*, 1998; Sorokin *et al.*, 2007). The high productivity is mainly driven by *Arthrospira* spp. and other cyanobacteria ( Melack & Kilham, 1974; Oduor & Schagerl, 2007). Cyanobacteria in lake lagoons only form algal mats in these lakes during rainy seasons ( Jones *et al.*, 1998; Muruga & Anyango, 2013; Krenitz & Shagerl, 2016). Reports indicate that *Ectothiorhodospira*, an anoxygenic phototrophic halophilic bacterium, and eukaryotes such as diatomic and green algae also play an essential part in primary production ( Matagi, 2004; Grant, 2006).

Many bacterial species have been isolated from extreme environments, and they frequently exhibit adaptations to optimal growth under the prevailing conditions ( Krulwich *et al.*, 2011; De Maayer *et al.*, 2014; Sorokin *et al.*, 2014b). Previously described isolates from Lake Magadi include the archaeal genera *Natronobacterium* and *Natronococcus* gen. nov. ( Tindall *et al.*, 1984) and *Natronobacterium magadii, Natrialba magadii* ( Kamekura *et al.*, 1997), bacterial species *Spirochaeta alkalica* sp. nov., *Spirochaeta Africana* ( Zhilina *et al.*, 1996), *Tindallia magadiensis* ( Kevbrin *et al.*, 1998) *, Halomonas magadii* ( Duckworth *et al.,* 2000) *, Amphibacillus fermentum* (renamed *Pelagirhabdus fermentum*) sp. nov., *Amphibacillus tropicus,* and *Halonatronum saccharophilum* ( Zhilina *et al.*, 2001) *, Methylonatrum kenyense* ( Sorokin *et al.*, 2007), *Euhalothece natronophila* ( Mikhodyuk *et al.*, 2008) and *Natranaerobaculum magadiense* ( Zavarzina *et al.*, 2013). Edwin *et al.* (2019) recovered 11 isolates affiliated with the cyanobacterial orders *Chroococcales*, *Oscillatoriales*, *Pleurocapsales*, and *Nostocales.* Recent studies have reported isolates affiliated with the genus *Bacillus*, *Alkalibacterium*, *Staphylococcus*, *Micrococcus*, *Halomonas*, and *Alkalilimnicola* ( Kiplimo *et al.*, 2019; Kipnyargis *et al.*, 2022). Orwa *et al.* (2020) recovered several fungal isolates affiliated with 18 different genera with *Aspergillus*, *Penicillium*, *Cladosporium*, *Phorma*, and *Acremonium* being dominant. Several studies have explored the microbial diversity in Lake Magadi using amplicons analysis targeting groups such as fungi ( Kambura *et al.*, 2016; Salano *et al.*, 2017; Mwirichia, 2022) or bacteria ( Kambura *et al.*, 2016).

A key ecological question is how microbial diversity changes with the fluctuating physicochemical conditions with seasons. We hypothesized that microbial communities within the lake shift in response to changes in the water chemistry over time. We predict that the communities in the brines are different from those in the open lake water. In this study, we explored the spatiotemporal variation in the microbial community over four months at different sites in Lake Magadi using 16S rRNA gene sequencing.

## Methods

### Sampling site and sampling criteria

**Figure 1.**
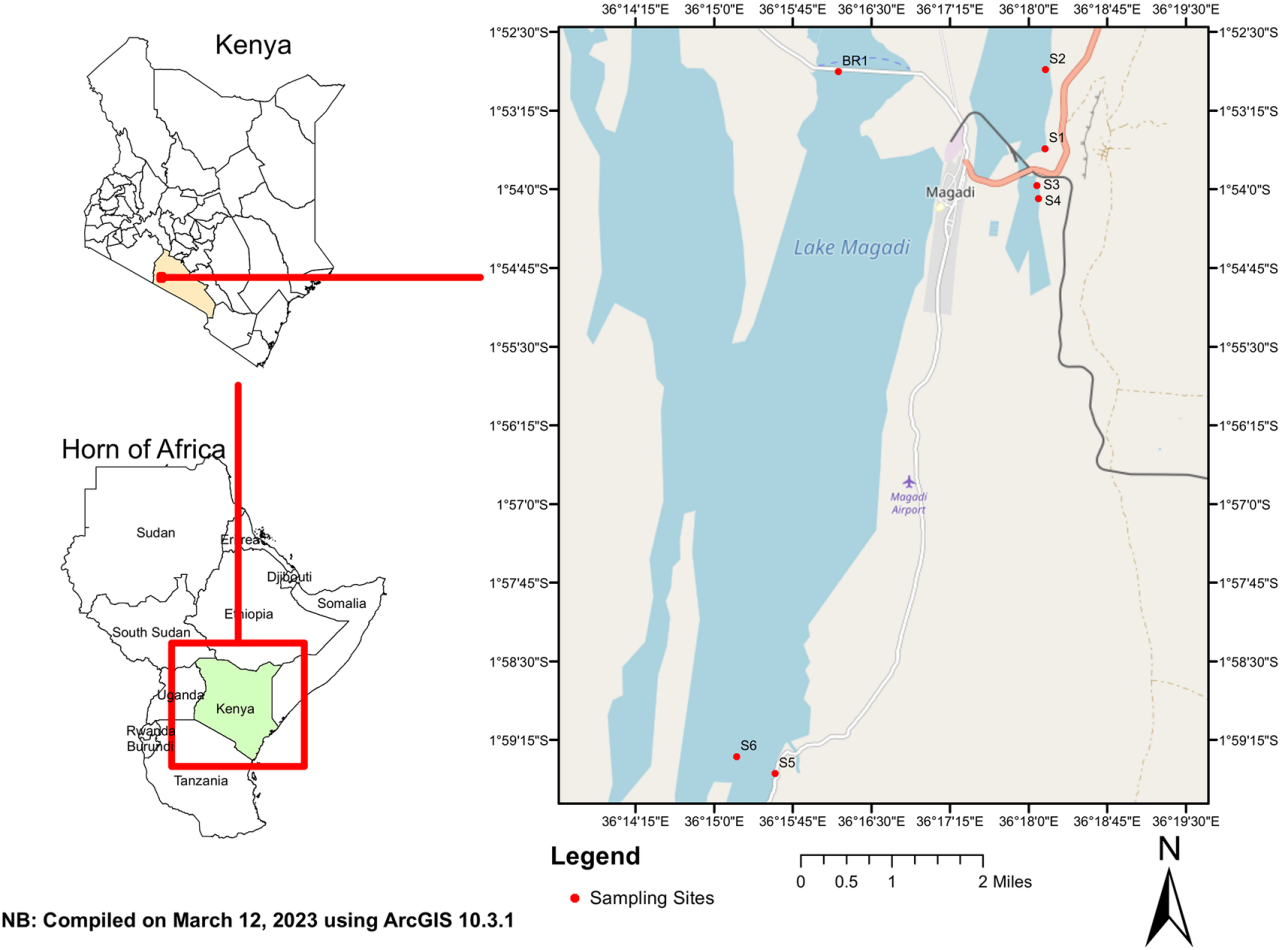
Map of Lake Magadi showing the sampling sites.

Sampling was done in hypersaline Lake Magadi, Kenya. It is located 1°43-2°00 S and 36°13-36°18E in an enclosed basin with an annual precipitation of 500 mm ( Behr & Röhricht, 2000). Lake Magadi is a relatively shallow water body that is fed by various hot springs distributed along the edges of the lake. The inflows have an influence on the lake volume and the water chemistry. Water samples were collected from different points in the lake including spring, brine, and open waters. Samples were collected from these sites in June, July, August, and September 2018 and the dry season lasted for all the sampling months, with June marking the start of the season and September being the driest month. The coordinates of the sampling sites were: S1 (1.891380 S; 36.302632 E), S2 (1.895020 S; 36.299372 E), S3 (1.900988 S; 36.301307 E), S4 (1.908460 S; 36.301996 E), S5 (1.991601 S; 36.258904E), S6 (1.975517 S; 36.236564 E) and BR1 (1.887908 S; 36.300855 E) ( Figure 1). S1 was composed of hot spring water, S2–S6 were composed of open waters, and BR1 was brine. Three sub-samples of 50 ml each were collected from each site and pooled into a composite sample. In addition, water samples for physicochemical analysis were collected. All samples were collected in sterile Conical Centrifuge tubes (Biologix, Shandong, China, Cat. No. 430829) and transported in a cool box.

### Analysis of physicochemical parameters

Water temperature, pH, total dissolved solids (TDS), and salinity measurements were recorded *in situ.* Water temperature, TDS, and salinity were measured using a VWR phenomenal handheld Meter (VWR, Atlanta, GA, USA, Model CO 3100H), while pH was measured using a Hanna Combo pH meter (Hanna Instruments, Nusafalau, Romania, Model HI-98128). In this case, about 100 ml of sample water was put in a sterile 400 ml glass beaker (Marienfeld, Germany, Cat. No. BR91236). A pre-calibrated meter was dipped in the sample and the readings were recorded. Water samples for dissolved P, K ^+^, NO _3_^-^, NH _4_^-^, Mg ^2+^, Na ^-^, Fe ^2-^, Ca ^2+^, SO _4_^2-^, Cl ^-^, and HCO _3_^-^ measurements were collected in sterile 500ml bottles and stored in a cool box for transportation to Crop Nutrition Laboratory Services (CNLS), Nairobi where analysis was done. Cations such as Ca, Mg, K, Na, Mn, Fe, Cu, Mo, B, Zn, and S were analyzed using atomic absorption spectrometry (AAS). At the same time, anion analysis was carried out using mass spectrometry.

### DNA extraction

Cell biomass for DNA extraction was obtained by centrifuging 50 ml of each water sample at 14,000 rpm for 20 minutes in an Eppendorf centrifuge (Eppendorf, Model 5415R, Cat. Z605212). The pellets were resuspended in 200 μl of a resuspension buffer (25% w/v sucrose (Sigma-Aldrich, Cat. No. S9378) in 50 mM Tris pH 8.5 (Sigma-Aldrich, Cat. No. 93352), and 50 mM EDTA; pH 8.0 (Sigma-Aldrich, Cat. No. 798681). To disrupt the cell wall of Gram positives, 2 μl of lysozyme (20 mg/ml) (Roche, Cat. No. 10837059001) and 10 μl of RNAse A (20 mg/ml) (Roche, Cat. No. 10109142001) were added and incubated at 37°C for 30 minutes. Cell lysis was achieved by the addition of 600 μl of a lysis buffer (1% SDS (Sigma-Aldrich, Cat. No. 8.17034) in 10 mM Tris pH 8.5 (Sigma-Aldrich) and 5 mM EDTA; pH 8.0 (Sigma-Aldrich). The samples were gently mixed with 10 μl of Proteinase K (20 mg/ml) (Sigma-Aldrich, Cat. No. 39450-01-6) and incubated at 65°C for 2 hours. DNA was recovered by adding an equal volume of chloroform (Sigma-Aldrich, Cat. No. C2432) followed by centrifugation at 13,200 rpm for 10 min at 4°C in an Eppendorf 5415R centrifuge. The aqueous layer was transferred into a new tube with 150 μl of sodium acetate (pH 5.2) (Sigma-Aldrich, Cat. No. S8750) and an equal volume of isopropyl alcohol (Sigma-Aldrich, Cat. No. 67-63-0). The contents were centrifuged at 13,200 rpm for 10 minutes and the DNA pellet was recovered by washing with 70% ethanol, air-dried for 15 minutes, and dissolved in 30 μl of nuclease-free water (Sigma-Aldrich, Cat. No. 7732-18-5). DNA quality was checked by running an aliquot of 2 μl in 1% agarose (Sigma-Aldrich, Cat. No. A9918) gel electrophoresis ( Orwa *et al*., 2020).

### Sequencing of the 16S rRNA amplicons

The V4 hypervariable region of the 16S rRNA genes was amplified using the universal primers for bacterial and archaeal primers 515F (5′-GTGCCAGCMGCCGCGGTAA-3′) and 806R (5′-GGACTACHVGGGTWTCTAAT-3′) ( Caporaso *et al*., 2012). Amplification was done using HotStarTaq Plus Master Mix Kit (Qiagen, USA) under the following cycling conditions: initial denaturation at 94°C for 3 minutes, followed by 35 cycles of denaturation at 94°C for 30 seconds, annealing at 53°C for 40 seconds and elongation at 72°C for 1 minute, after which a final elongation step at 72°C for 5 minutes was performed. Three independent PCR reactions were performed per sample and pooled in equimolar amounts. The PCR products were then checked in a 2% agarose gel. The sample was purified using calibrated Ampure XP beads (Beckman Coulter, Inc., IN, USA). DNA libraries were prepared using Illumina TruSeq DNA libraries (Illumina, Inc., San Diego, CA, United States) and sequencing was performed at MR DNA (Shallowater, TX, USA) on a MiSeq platform (2 × 300 bp) following the guidelines of the manufacturer (Illumina Inc.).

### Sequence processing and taxonomic assignment

The Q25 sequence data derived from MiSeq sequencing was processed using the MR DNA ribosomal and functional gene analysis pipeline (MR DNA, Shallowater, TX). Sequences were depleted of primers, reads <250 bp, and ambiguous base calls were removed. The reads were quality-filtered using a maximum expected error threshold of 1.0. Sequences were further processed using VSEARCH v2.14 ( Rognes *et al.*, 2016). This included sorting and size-filtering of the paired reads to ≥300 bp (--sortbylength --minseqlength 300) and dereplication (--derep_fulllength). The sequences were then denoised and evaluated for potential chimeric sequences using UCHIME package v.11. ( Edgar *et al.*, 2011). Representative operational taxonomic units (OTUs) were picked *de novo* using VSEARCH v2.14 ( Rognes *et al.*, 2016), and assigned taxonomy using BLAST searches against the SILVA v132 rRNA reference database ( Quast *et al.*, 2012). A sequence identity cutoff of 97% was used to pick OTUs from the quality-filtered, denoised, non-chimeric sequences. Eukaryotic sequences were filtered from the dataset using the script filter_otu_table.py. in QIIME v1.90 ( Caporaso *et al.*, 2010b).

The Illumina raw reads for the 16S rRNA gene sequences were deposited in the Sequence Read Archive (SRA) of NCBI under the accession numbers PRJNA962270 ( Kipnyargis *et al*., 2023b).

### Microbial community analysis

Sequences with assigned taxonomy were aligned using PyNast ( Caporaso *et al.*, 2010a), and a phylogenetic tree was constructed using FastTree v2.1.7 ( Price *et al.*, 2010). The alpha diversity indices (Chao1, abundance-based coverage estimator (ACE), Simpson, Shannon, Fisher’s alpha, Pielou’s evenness, and Good’s coverage) were calculated with QIIME v1.90 (Caporaso *et al.*, 2010) using *alpha_rarefaction.py* employing the same level of surveying effort (37,000 per sample based on the lowest sample count). All subsequent steps were analyzed in R software v4.2.0 ( R Core Team, 2020) and RStudio v1.1.456 ( RStudio Team, 2020). The results of all statistical tests were regarded as significant if p ≤ 0.05. To compare the (dis) similarity of OTU compositions between communities the OTU abundance table was standardized using decostand (method = “hellinger”). Hierarchical cluster analysis was performed using hclust in R software v4.2.0 ( R Core Team, 2020) (method = “average”). The heatmap was created using JColorGrid v1.86 ( Joachimiak *et al.*, 2006).

The OTU network generated in QIIME was filtered using an edge cut-off of 0.001 and visualized in Cytoscape v3.9.1 ( Otasek *et al.*, 2019) in an “edge-weighted spring-embedded layout”. In this case, sampling sites were used as source nodes and bacterial families as target nodes. Redundancy analysis (RDA), based on Bray dissimilarity was used to test the correlation between the physicochemical parameters and the microbial community at the genus level. This was done using the *Microeco* package v0.15.0 ( Liu *et al.*, 2021) and plotted using the package *Pheatmap* in R.

To assess the beta diversity of microbial communities, a non-metric multidimensional scaling (NMDS) was performed using Bray-Curtis dissimilarities with the script compare_categories.py. test and weighted UniFrac distance matrix ( Lozupone & Knight, 2005) as input using the Vegan package in R ( Bray & Curtis, 1957; Oksanen, 2015).

## Results

### Physicochemical properties of the sampling sites

One of the objectives of this study was to investigate the change in water chemistry over time. It has been established that physicochemical factors play a critical role in shaping the structural composition of microbial communities in an ecosystem. Samples from site S1 (hot spring water) exhibited lower concentrations of the various ions and cations as compared to the other samples. The water temperature ranged from 27°C to 38.7°C (average 33.7°C). The pH of the water was alkaline, ranging from 9.8 (S6_June) to 11.5 (BR1_June) recording the highest pH value of 11.5. The major water cations were Na ^+^ (10,300–160,000 ppm) and K ^+^ (131–4,280 ppm), and the major anions were HCO _3_^−^ (15,400–277,000ppm) and Cl ^−^ (4,050–102,000 mg/L). Phosphorus levels ranged from 2.38–108 ppm, while magnesium and calcium levels were low, ranging from 0.02–16.1 and 0.05–127 ppm, respectively. The total dissolved solids (TDS) ranged from 27.1–153.5 ppm ( Table 1).

**Table 1.** Physicochemical characteristics of the water samples collected from Lake Magadi. TDS total dissolved solids, SAR sodium absorption ratio. The samples are denoted as S1 to S6, while BR1 represents the brine sample.

Sample	S1_June	S1_Sep	S2_June	S2_July	S2_Sep	S3_June	S3_July	S3_Aug	S3_Sep	S4_June	S4_July	S4_Aug	S4_Sep	S5_June	S5_July	S5_Aug	S5_Sep	S6_June	S6_July	S6_Aug	BR1_June	BR1_Sep
**pH**	10.5	10.4	10.6	10.2	10.7	10.5	10.5	11.3	10.4	10.5	10.5	11.1	10.9	10.2	9.9	10.3	10.2	10.3	9.8	10.4	11.5	11.2
**Temp. (°C) **	35.6	35.6	35.1	32.8	37.2	38.7	27	34.6	35.6	37.3	27	33.6	37.8	34.2	32.7	29.8	32.8	32.5	30.2	29.2	38.3	34.8
**TDS (ppm)**	27.1	27.1	137.6	143.9	145	114.1	135.4	139.2	153.5	110.7	134.6	139.8	143.3	46	45.7	45.7	42.9	104.9	118.1	83.9	136	134.8
**P (ppm)**	2.38	7.77	58.4	78	117	27.4	77.2	108	69.8	26.4	63.8	107	105	3.96	2.39	5.79	10.6	16.2	21.9	4.91	81.1	92.8
**K (ppm)**	131	365	2,300	2,700	4,280	1,220	2,430	3,300	2,560	1,130	1,960	3,270	2,370	201	190	280	378	697	697	513	3,410	3,210
**NO _3_ (ppm)**	0.89	9.03	1.81	0.01	5.98	0.01	0.01	0.2	4.96	0.01	0.01	0.01	6.99	4.56	0.01	0.19	8.5	32	0.01	0.01	0.01	7.44
**NH _4_ (ppm)**	0.02	0.69	0.072	0.2	0.98	0.017	0.082	0.013	0.53	0.033	0.01	0.056	0.86	0.076	0.076	1.04	1.52	0.01	0.16	0.59	0.47	0.79
**Mg (ppm)**	0.026	5.86	2.49	6.58	2.63	2.82	8.2	0.02	4.76	2.41	6.31	0.3	2.31	0.81	4.46	0.02	2.57	3.89	6.02	0.02	1.44	16.1
**Mn (ppm) **	0.06	0.01	0.045	0.066	0.01	0.076	0.23	0.01	0.019	0.15	0.13	0.11	0.01	0.01	0.01	0.01	0.01	0.24	0.74	0.15	0.028	0.53
**S (ppm)**	39.9	132	548	629	1010	324	708	958	710	304	585	973	949	97.2	90.7	71.4	182	213	271	178	845	974
**Cu (ppm)**	0.01	0.15	0.01	0.01	0.15	0.01	0.01	0.22	0.086	0.01	0.01	0.01	0.098	0.01	0.01	0.01	0.11	0.01	0.01	0.068	0.01	0.4
**B (ppm) **	6.8	14.6	103	126	197	55.7	126	184	116	52	108	184	169	13	12.4	11.2	20.4	37.8	43.6	28.2	147	160
**Zn (ppm) **	0.01	0.01	0.01	0.01	0.01	0.01	0.01	0.01	0.01	0.01	0.01	0.01	0.01	0.01	0.01	0.01	0.01	0.01	0.01	0.01	0.33	0.29
**Na (ppm) **	10,300	22,900	93,400	114,000	143,000	63,900	120,000	121,000	100,000	58,800	100,000	118,000	104,000	18,300	17,700	16,000	30,800	58,800	70,500	36,000	155,000	160,000
**Fe (ppm)**	2.79	0.53	0.01	1.34	0.01	2.13	9.63	0.91	0.92	2.42	3.17	1.59	0.17	0.25	2.5	0.01	0.25	4.1	14.7	3.82	0.86	0.49
**Ca (ppm)**	0.05	3.79	8.52	10.9	0.34	12	16.4	0.2	7.43	10.8	9.23	1.13	6.54	0.05	2.94	0.05	2.72	13.8	15.8	0.05	2.15	127
**SO _4_ (ppm)**	120	395	1,640	1,880	3,030	971	2,120	2,870	2,130	911	1,750	2,920	2,840	291	272	214	545	638	812	533	2,530	2,920
**Mo (ppm) **	0.067	0.25	1.66	2.49	3.82	1.03	2.43	4.99	2.23	0.83	1.74	4.5	3.09	0.048	0.23	2.09	0.24	0.29	0.4	1.68	2.37	2.79
**Cl (ppm)**	4,050	3,720	52,300	77,400	102,000	30,700	72,300	99,800	74,200	30,200	60,500	93,100	62,500	7,910	8,620	7,680	6,740	24,100	32,500	16,700	93,500	99,000
**NO _3_N (ppm)**	0.2	2.04	0.41	0.01	1.35	0.01	0.01	0.045	1.12	0.01	0.01	0.01	1.58	1.03	0.01	0.043	1.92	7.23	0.01	0.01	0.01	1.68
**HCO _3_ (ppm)**	17,300	15,400	145,000	205,000	213,000	94,300	229,000	242,000	143,000	94,700	196,000	233,000	165,000	30,100	30,200	29,300	25,300	98,200	141,000	70,600	256,000	277,000
**Si (ppm)**	66.2	114	447	568	786	282	569	861	590	284	458	867	760	89.9	94.9	194	107	106	197	287	595	696
**SiO _2_ (ppm)**	142	244	956	1,220	1,680	603	1,220	1,840	1,260	608	980	1,850	1,630	192	203	415	229	227	421	614	1,270	1,490
**SAR (ppm)**	9,310	1,720	7,230	6,730	18,200	4,310	6,040	69,000	7,050	4,210	6,220	25,500	8,900	4,290	1,520	15,300	3,220	3,600	3,830	34,400	20,100	3,550
**CaCO _3_ (ppm)**	0.23	33.5	31.5	54.2	11.6	41.6	74.6	0.58	38.1	36.9	48.9	4.05	25.8	3.45	25.6	0.21	17.3	50.4	64.2	0.21	11.3	384

### Sequence analysis and diversity studies

After quality filtering, denoising, and removal of potential chimeras and non-bacterial sequences, approximately 3,197,447 high-quality sequences with an average read length of 525 bp were obtained from the entire dataset. The number of sequences per sample varied from 37,406 (sample S5_Jun) to 285,085 (sample BR1_Sep) with an average value of 121,603 sequences. The number of OTUs per sample ranged from 852 (sample S3_July) to 2,024 (sample S5_Sep) ( Table 2). Sequencing generated a total of 4,837 OTUs distributed in the domain Bacteria (3,802 OTUs) and Archaea (1,035 OTUs). Overall, most OTUs were found in the open waters samples of S5, while S4 had the least number of OTUs. The distribution of shared OTUs based on the month of sampling is shown in *Extended data*, Supplementary Figure 1 ( Kipnyargis *et al*., 2023a).

**Table 2.** Number of sequences generated, OTUs, and diversity indices of 22 sampling sites in Lake Magadi. OTUs, operational taxonomic units; ACE, abundance-based coverage estimator.

Sample	Number of Sequences	OTUS	Chao1	Ace	Simpson	Observed species	Shannon	Fisher alpha	Goods coverage
BR1_June	149,051	1,416	357.30	414.77	0.93	148	5.28	53.40	0.89
S1_June	191,597	1,089	171.50	175.11	0.93	89	4.63	25.63	0.94
S2_June	174,894	1,267	180.77	181.63	0.88	94	4.32	27.66	0.94
S3_June	139,647	1,513	251.65	319.48	0.94	131	5.15	44.51	0.91
S4_June	130,649	1,514	306.14	397.09	0.92	140	5.00	49.13	0.89
S5_June	37,406	1,221	363.23	452.68	0.96	209	6.16	92.00	0.85
S6_June	116,475	1,665	350.70	385.77	0.96	179	5.90	71.63	0.87
S2_July	177,773	1,259	301.33	300.15	0.91	98	4.49	29.32	0.92
S3_July	177,827	852	171.50	162.62	0.66	69	2.86	18.11	0.95
S4_July	156,660	1,167	166.62	189.02	0.90	87	4.31	24.84	0.94
S5_July	109,083	1,917	435.14	487.08	0.97	231	6.54	108.85	0.83
S6_July	103,156	1,149	181.32	192.37	0.86	106	4.19	32.76	0.93
S3_Aug	177,142	874	91.91	107.34	0.79	55	3.20	13.39	0.96
S4_Aug	172,666	934	175.86	143.57	0.84	70	3.70	18.46	0.95
S5_Aug	83,183	1,815	572.10	579.97	0.97	222	6.28	101.75	0.82
S6_Aug	64,022	1,732	497.95	457.95	0.98	200	6.57	85.59	0.86
BR1_Sep	285,085	1,710	323.50	356.33	0.95	178	5.75	71.00	0.88
S1_Sep	180,065	1,583	341.16	436.97	0.95	177	5.77	70.38	0.87
S2_Sep	170,143	1,114	184.23	213.10	0.89	90	4.34	26.03	0.94
S3_Sep	149,323	1,124	149.37	174.86	0.75	90	3.50	26.03	0.94
S4_Sep	151,058	956	185.17	135.22	0.80	68	3.26	17.76	0.95
S5_Sep	100,542	2,024	594.50	641.88	0.98	248	6.85	123.09	0.81

### Alpha diversity studies

The values of the good’s coverage estimator ranged from 81% (S5_Sep) and 96% (S3_Aug) suggesting that the sequencing process captured a significant number of dominant communities. Within the open water samples (S2–S6), site 5 samples collected across the seasons had the highest alpha diversity indices suggesting that S5 had the highest species richness and diversity. S3_Aug samples (open waters) had the lowest alpha diversity indices. Within the hot spring samples (S1), S1 samples collected in September had the highest species richness and diversity. Within the brine samples (BR1), Br1 samples collected in September had the highest species diversity and richness ( Table 2).

**Figure 2.**
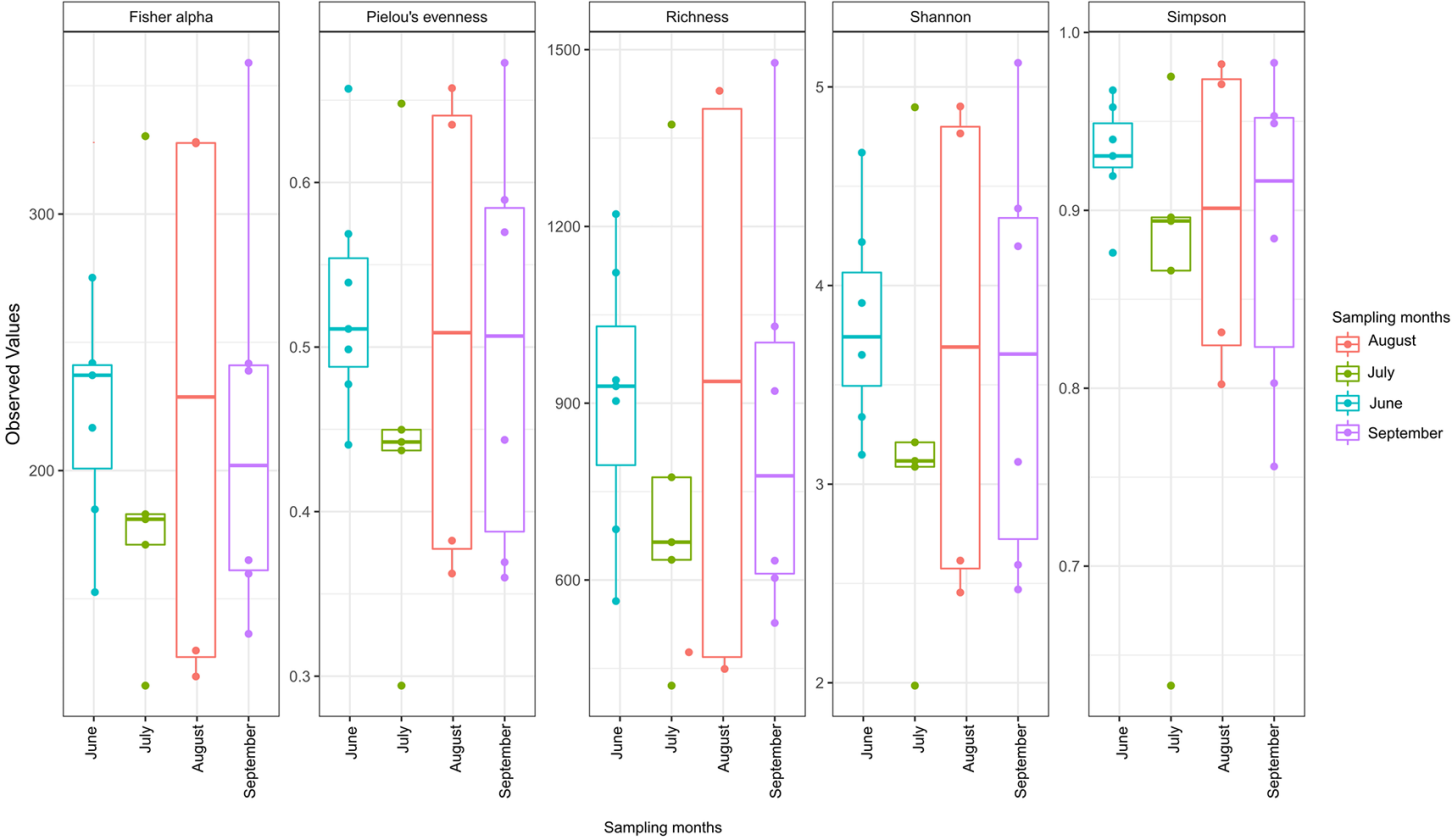
Alpha diversity plots of OTU richness among different sampling months.

The alpha diversity indices showed that high microbial diversity was recorded in August, followed by September, June, and July in that order ( Figure 2).

### Beta diversity studies

**Figure 3.**
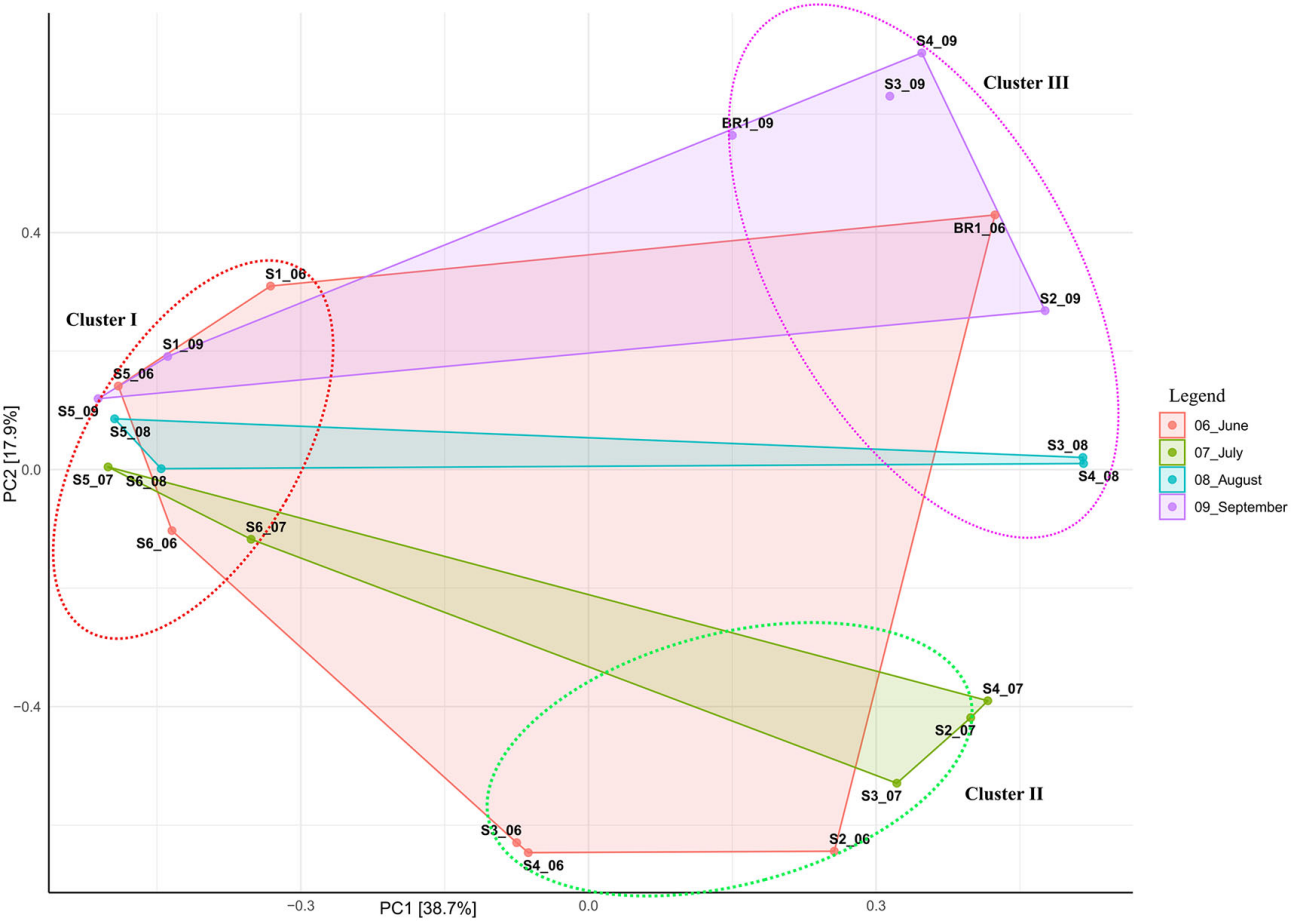
Principal component analysis (PCA) ordination of differences in microbial communities from sampling sites and sampling months.

Beta diversity ordination based on Bray-Curtis dissimilarity showed that samples (except hot spring and brine samples) did not cluster based on the sampling site. Overall, all samples clustered together based on salinity and alkalinity, indicating the impact of these elements on the structure of the bacterial and archaeal communities ( Figure 3; Table 1). The principal component (PCA) analysis showed that the first (PC1) and second (PC2) axes described 38.7% and 17.9% of the variance in microbial communities, respectively. Accordingly, samples were clustered into three distinct groups based on alkalinity and salinity. Low alkalinity and salinity samples (pH 9.8 – 10.5; 10,300 ppm – 70,500 ppm) formed cluster I with nine samples (S1_06, S1_09, S5_06, S5_09, S5_08, S5_07, S6_08, S6_06, and S6_07). Moderately alkaline and saline samples (pH 10.5 – 10.6; 63, 900 ppm – 100,000 ppm) formed cluster II with six samples (S3_06, S4_06, S2_06, S3_07, S2_07, and S4_07). Highly alkaline and saline samples (pH 10.7-11.5; >100,000 ppm) formed cluster III consisting of six samples (Br1_06; 155,000 ppm, Br1_09; 160,000 ppm, S4_09; 104,000 ppm, S2_09; 143,000 ppm, S3_08; 121,000 ppm, and S4_08; 118,000 ppm).

### Taxonomic composition and structure

**Figure 4.**
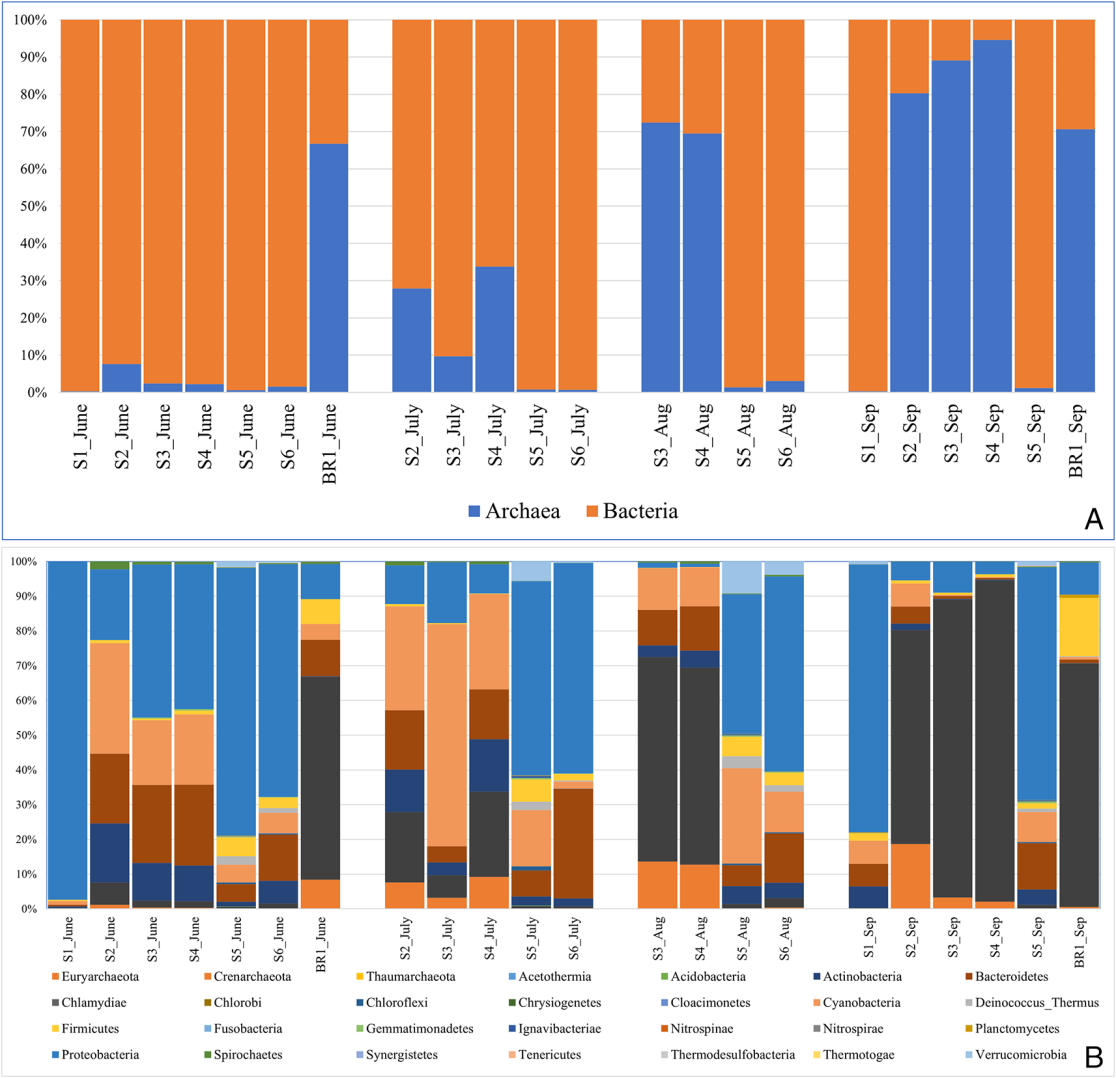
Relative abundance and composition of the microbial community taxa based on sampling sites and month of sampling. (A) The proportion of Domains bacteria and archaea across the sampling sites and months. (B) Relative abundance of the most popular bacterial and archaeal phyla across the sampling sites and the sampling months.

The proportion of bacteria to archaea varied by season and sampling month ( Figure 4). The results indicate that the archaeal population increased as the ion concentration increased while bacteria abundance was higher where the ion concentration was lower (sites 1, 4, and 5) ( Table 1). In hot spring water (S1), archaea abundance was the lowest while bacterial abundance was the highest. Within open water samples (S2–S6), S4 had the highest abundance of archaea, while S5 had the highest proportion of bacteria. Within the brine (BR1), the archaea proportion was relatively higher than the bacterial communities. From June to September 2018, bacterial abundance decreased while archaeal abundance increased ( Figure 4A).

The bacterial reads were distributed across 25 phyla, 107 orders, 225 families, and 545 genera. The results revealed that the most abundant bacterial phyla across the sampling sites and the four months of sampling included Proteobacteria (35% of all the reads), Cyanobacteria (14.2%), Bacteroidetes (10.9%), Actinobacteria (5.2%), Firmicutes (2.7%), Verrumicrobia (1.1%). Deinococcus-Thermus (0.6%), Spirochaetes (0.4%), and Chloroflexi (0.1%) were detected in lower abundances ( Figure 4B). At the genus level, the dominant bacterial genera (> 1% of all sequences across all samples) were *Euhalothece* (10.3%), *Rhodobaca* (9.6%), *Idiomarina* (5.8%), *Rhodothermus* (3.0%), *Roseinatronobacter* (2.4%), *Nocardioides* (2.3%), *Gracilimonas* (2.2%), *Halomonas* (2.0%), *Lewinella* (1.9%), *Synechococcus* (1.8%), *Aliidiomarina* (1.8%), *Nitriliruptor* (1.7%), *Thioalkalivibrio* (1.7%), *Salinibacter* (1.4%), *Alkalimonas* (1.25%), *Chelatococcus* (1.4%), and *Rhodovulum* (1.4%). Others included: *Cytophaga* (0.9%), *Natronocella* (0.9%), *Thiohalomonas* (0.9%), *Euzebya* (0.8%), *Paracoccus* (0.8%), and *Luteolibacter* (0.8%). The abundance of bacterial genera was higher in the sampling site S5 (25.3%) followed by S6 with 20.1%, S3 (14.5%), S1 and S4 with 12.8% each, and brine sample BR1 with 3.9% abundance in that order. In terms of the sampling month, June had the highest bacterial abundance with 39.5% followed by July (27%), September (16.8%), and August (16.2%) in that order.

The archaeal reads were affiliated to three phyla (Euryachaeota, Crenarchaeota, and Thaumarchaeota) ( Figure 4B), 14 orders, 20 families, and 62 genera. The dominant Phylum was Euryachaeota (87% of all Archaeal samples), with its dominant genera (>1% of all sequences across all samples) being *Halorubrum* (18.3%), *Salinarchaeum* (5.4%), and *Haloterrigena* (1.3%). Other genera included *Methanomassiliicoccus* (0.6%), *Palaeococcus* (0.4%), *Halovenus* (0.3%), *Thermococcus* (0.3%), *Haladaptatus* (0.3%), *Halorientalis* (0.3%), *Methanobrevibacter* (0.2%), *Natronomonas* (0.2%), *Halohasta* (0.2%), *Haloquadratum* (0.1%), and *Methanobacterium* (0.1%). The abundance of archaeal genera was higher in S3 (27.2%) followed by brine site BR1 with 21.6% abundance. S1 and S6 had the least archaeal abundance with 0.08 and 0.8%, respectively. In terms of the sampling month, September had the highest archaeal abundance with 53% followed by August (23%), June (12.7%), and July (11.4%).

**Figure 5.**
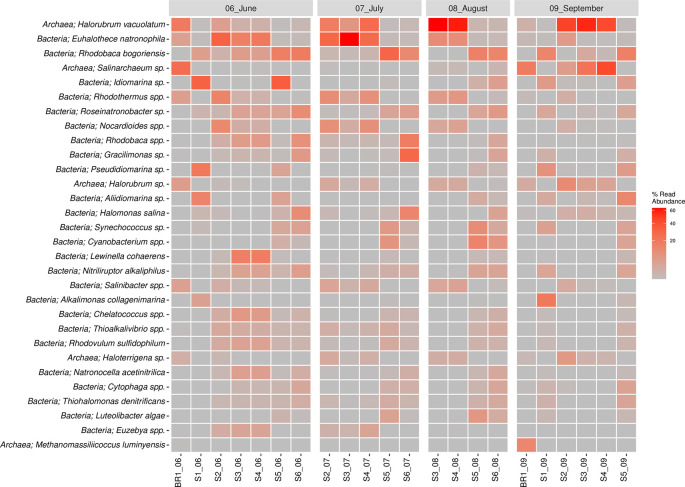
Read abundance (%) of the top 30 species across samples.

The bacterial species composition (>1%) included *Euhalothece* spp. (10.3%), *Rhodobaca* spp. (9.6%), *Idiomarina* spp. (5.8%), *Rhodothermus* spp. (3.0%), *Roseinatronobacter* spp. (2.4%), *Nocardioides* spp. (2.3%), *Gracilimonas* spp. (2.2%), *Halomonas* sp. (2%), *Lewinella* (1.9%), *Synechococcus* spp. (1.8%), *Cyanobacterium* spp. (1.8%), *Aliidiomarina* spp. (1.7%), *Nitriliruptor* spp. (1,7%), *Thioalkalivibrio* spp. (1.7%), *Salinibacter* spp. (1.4%), *Alkalimonas* spp. (1.2%), *Chelatococcus* spp. (1.1%), and *Rhodovulum* spp. (1.1%). The *Euhalothece natrophila* species were abundant in June, July, and August, except in sites S5 and S6 across all seasons. *Rhodobac*a *bogoriensis* was largely sampled in June and site S6 in July and August 2018. *Idiomarina* spp. were largely concentrated in June, particularly in sites S1 and S5, whereas *Rhodovulum* spp. were sampled across all seasons. *Lewinella coherens* were sampled in June mostly in sites S3 and S4. On the other hand, *Halorubrum* spp. (18.3%), *Salinarchaeum* spp. (5.3%), *Haloterrigena* spp. (1.3%), *Methanomassiliicoccus* spp. (0.7%), and *Palaeococcus* spp. (0.5%) were the major species in the Archaeal Domain. *Idiomarina vacuolatum* was sampled across all the sampling seasons but its abundance varied across the sampling sites. *Halorubrum vacuolatum* was mainly sampled in August (S1 and S2) and September (S3, S4, and S5). *Salinarchaeum* sp. was mainly sampled in September, while *Haloterrigena* spp. was sampled across the seasons and sites, though in low proportions. The top 30 most abundant species of bacteria and archaea are shown in Figure 5. Overall, *Halorubrum* spp. was the most abundant species sampled followed by *Euhalothece* spp. and *Rhodobaca* spp. ( *Extended data,* Supplementary Figure 2 ( Kipnyargis *et al*., 2023a)).

**Figure 6.**
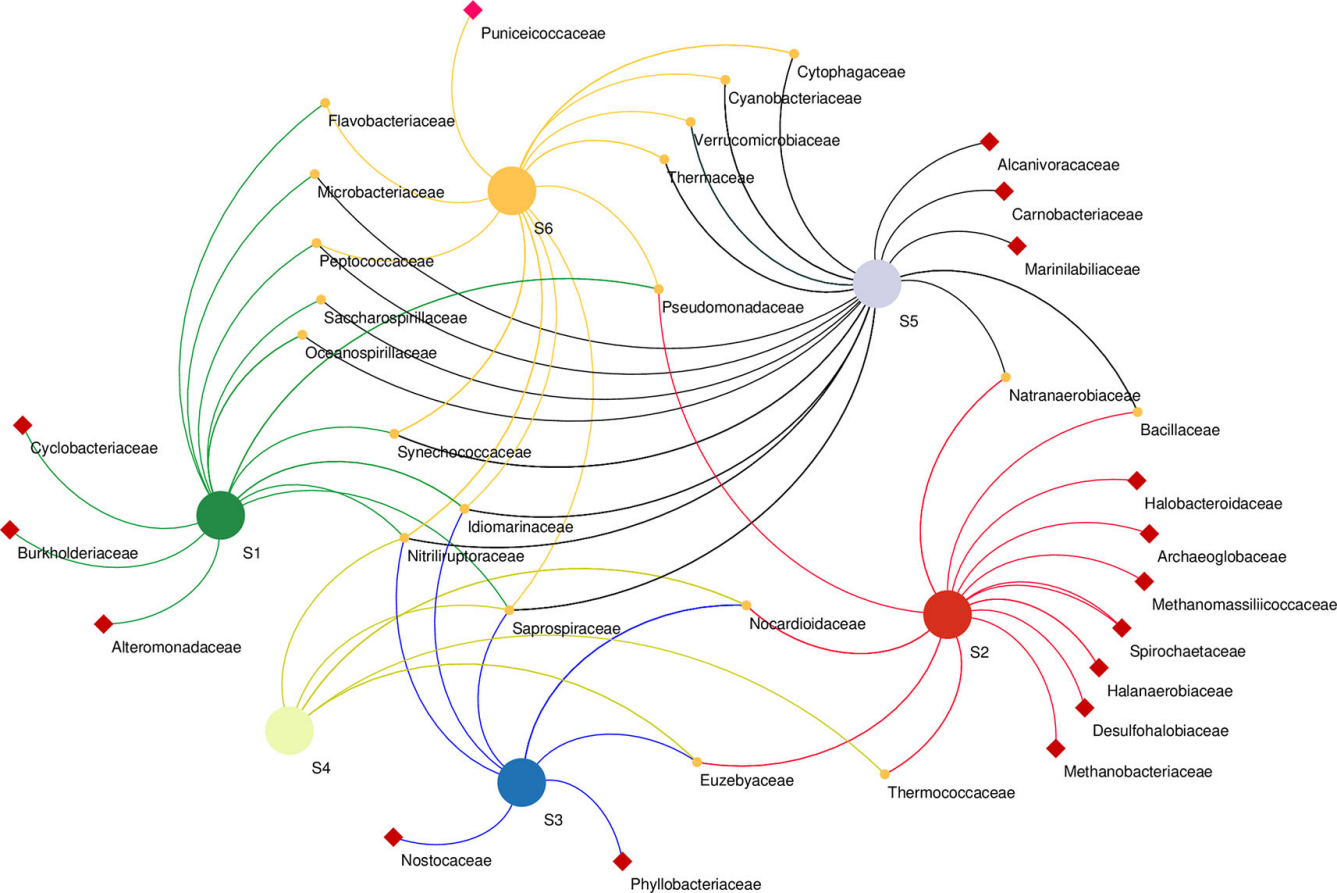
Network analysis of microbial community at Family level based on sampling sites. Samples marked with red squares indicate their exclusive site of isolation.

Microbial co-occurrence network analysis at the family level revealed that bacterial members of the family Cyclobacteriaceae, Burkholderiaceae, and Alteromonadaceae were unique to the S1 sampling site. Bacterial members of Halobacteroidaceae, Spirochaetaceae, halanaerobiaceae, and desulfohalobiaceae, as well as Archaeal members of Archaeoglobaceae and Methanobacteriaceae, were found exclusively in the S2 sampling site. Phyllobacteriaceae and Nostocaceae were unique to S3, while Alcanivoracaceae, Carnobacteriaceae, and Marinilabiliaceae bacteria were found in S5 only. Unique to S6 were the bacterial Puniceicoccaceae family. The highest number of co-shared families was found between S5 and S1 co-sharing eight families, whereas Bacilaceae and Natranaerobiaceae were found in S5 and S2, and S6 and S2 co-shared only one family (Pseudomonadaceae) ( Figure 6).

### Physicochemical drivers of bacterial and archaeal community structure

**Figure 7.**
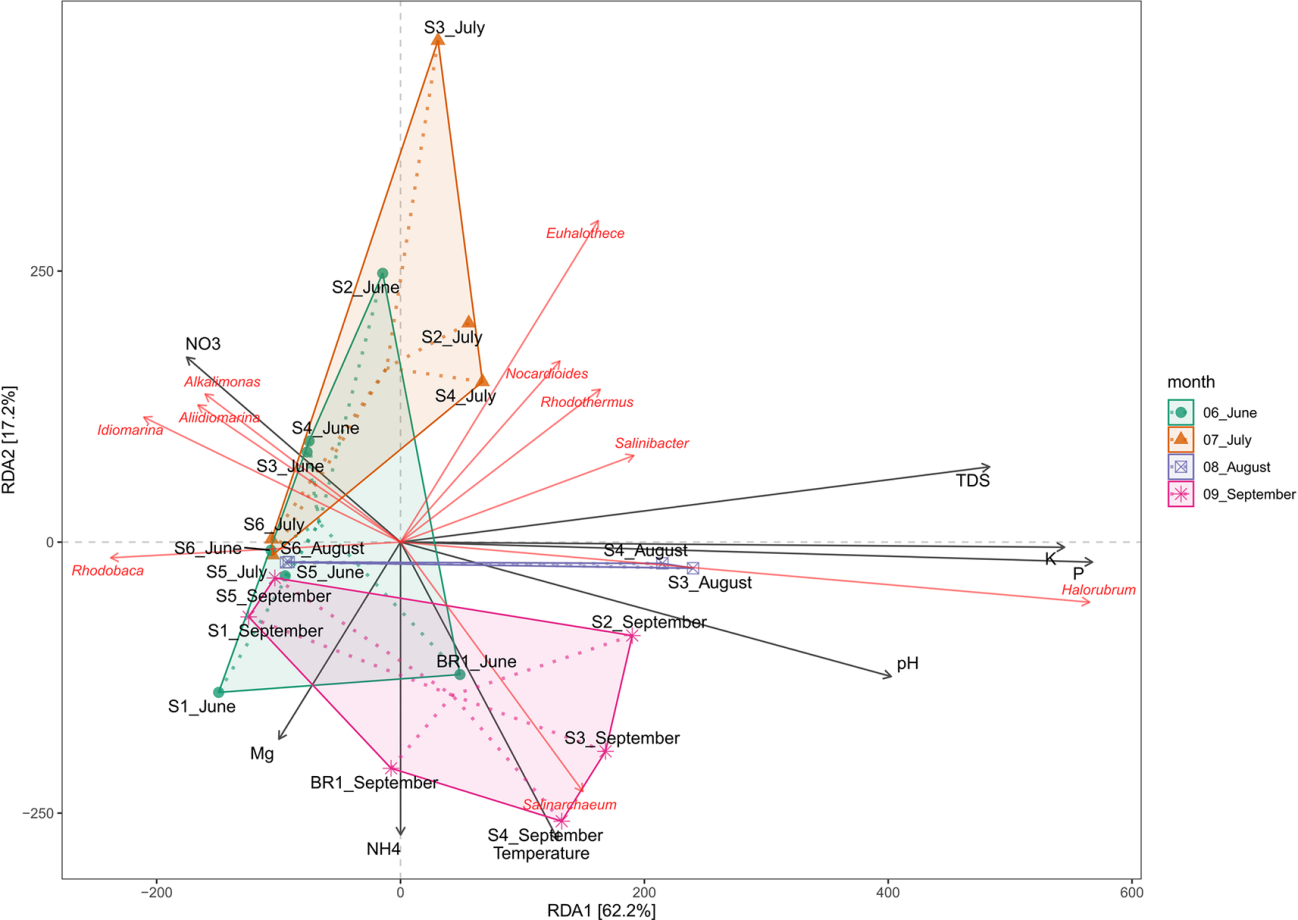
Redundancy Analysis (RDA) ordination showing the relationships between the physicochemical factors and the dominant genera in Lake Magadi. Samples corresponding to their sampling month are indicated, while genera are written in red, and environmental variables are indicated by arrows. TDS corresponds to total dissolved solids.

Redundancy analysis (RDA) was used to assess the effect of water chemistry on microbial community structure in Lake Magadi. The results reveal that changes in the physicochemical parameters influenced the microbial communities in the lake ( *Extended data*, Supplementary Table 2 ( Kipnyargis *et al*., 2023a)). The RDA explained 62.2% and 17.2% of the variation in the first (RDA1) and the second (RDA2) axes, respectively ( Figure 7).

The results demonstrated that temperature, pH, P, K, NO _3_^-^, and TDS significantly influenced the microbial community structure. Generally, members of genera *Nocardiodes*, *Rhodothermus*, *Haloterrigena*, *Methanomasiliicoccus*, *Halorubrum*, *Palaeococcus*, *Nocardioides*, *Salinarcheum*, *Salinibacter*, and *Euhalothece* spp. had a wide range of adaptability. Conversely, *Synechococcus*, *Thioalkalivibrio*, *Cyanobacterium*, *Rhodovulum*, *Lewinella*, *Idiomarina*, *Pseudidiomarina*, *Chelatococcus*, *Aliidiomarina*, and *Alkalimonas* spp. were least influenced by the tested physicochemical factors ( *Extended data*, Supplementary Figure 3 ( Kipnyargis *et al*., 2023a)). Notably, *Halorubrum* and *Haloterrigena* spp. were positively correlated with P and K (R ^2^ = 0.66, p < 0.001), but negatively correlated with Mn and CO _3_^2-^. pH and NH4 ^+^ appear to positively correlate with the structure of the members of the genus *Salinarcheum* (R ^2^ = 0.245; p < 0.004), but negatively correlated with NO _3_^-^. Members of *Alkalimonas*, *Idiomarina,* and *Aliidiomarina* spp. were positively correlated with NO _3_^-^ (R ^2^ = 0.049, p < 0.210), but negatively correlated with all other tested parameters. Members of *Nocardiodes*, *Rhodothermus*, *Salinarcheum*, *Salinibacter,* and *Euhalothece* spp. were positively correlated with total dissolved solids (TDS), alkalinity, salinity, CO _3_^2+^, and NH _4_^+^ (R ^2^ = 0.606, p < 0.001), but negatively correlated with Mg ^2+^, Mn, and NO _3_^-^. On the other hand, Mn, temperature CO _3_^2-^, and NH _4_^+^ negatively affect the structure of *Rhodobaca.*

## Discussion

We explored the structure and composition of microbial communities based on the seasonality and physicochemical parameters of Lake Magadi. The physicochemical parameters revealed high concentrations of sodium salts, HCO _3_^-^, SO _4_^2-^, pH values of 9.8–11.5, temperatures of 27–38°C, and low concentrations of Ca ^2+^, Mg ^2+^, and Cu ^2+^. These findings were consistent with previous reports indicating that soda lakes are characterized by moderate to high temperatures, high concentrations of HCO _3_^−^/CO _3_^2−^, and reduced concentrations of Ca ^2+^ and Mg ^2+^ ( Sorokin *et al.*, 2014a; Vavourakis *et al.*, 2018). However, total dissolved solids (TDS) ranged from 27 ppm (0.02g/L) to 143 ppm (0.143g/L), a situation that is lower than other soda lakes ( Taher, 1999; Hosam *et al.*, 2017; Pérez & Chebude, 2017). Sulfate concentration (120–3,030 ppm) was similar to most soda lakes in East Africa ( Lameck *et al*., 2023), but higher than the concentration reported in Lake Lancago in Qinghai-Tibet ( Wang *et al*., 2022). Comparatively lower sulfate concentrations have been reported from lakes Sidi Ameur and Himalatt (Algeria) ( Boutaiba *et al.*, 2012) and Lake Hamra in Egypt ( Mahmoud *et al.*, 2024). The concentrations of the measured elements (except pH) were variable from site to site and fluctuated with time, indicating that the lake chemistry is constantly changing in its constituent elements. A high and stable pH recorded in Lake Magadi is due to high amounts of carbonates that maintain a constant pH in soda lake ecosystems ( Simachew *et al.*, 2016). It is postulated that Ca ^2+^ and Mg ^2+^ precipitate as insoluble carbonates due to high evaporation rates in these ecosystems. As a result, an alkaline brine with Na ^+^, Cl ^−^, and HCO _3_^−^/CO _3_^2−^ accumulates as main ions. The shift in CO _2_/HCO _3_^−^/CO _3_^2−^ equilibrium towards CO _3_^2−^, leads to the formation of a soda (Na _2_CO _3_) lake with pH values of over 10.0 ( Grant and Jones, 2016).

A high number of OTUs was detected for the domain Bacteria with 3,802 OTUs while Archaea had 1,035 OTUs. Bacterial diversity was dominated by the phyla Proteobacteria (35% of all the reads), Cyanobacteria (14.2%), Bacteroidetes (10.9%), Actinobacteria (5.2%), Firmicutes (2.7%), Verrumicrobia (1.1%). On the other hand, Deinococcus-Thermus (0.6%), Spirochaetes (0.4%), and Chloroflexi (0.1%) were detected in lower abundances in Lake Magadi. Similar results have been shown from soda ecosystems such as Solar saltern in Tunisia ( Menasria *et al.*, 2019), lake Chott El Jerid ( Abdallah *et al.*, 2018), hot springs of Lake Magadi ( Kambura *et al.*, 2016), and lakes Sonachi, Magadi, Elmenteita, and Bogoria in Kenya ( Mwirichia, 2022). Members of the phylum Proteobacteria were the most dominant group across all the time points and sampling sites. The role of members of Proteobacteria such as *Burkholderiaceae* is to decompose recalcitrant organic matter while others like *Beijerinckiaceae* fix atmospheric nitrogen ( Li *et al.*, 2012). The phylum Cyanobacteria, the major contributor to nitrogen fixation in soda lakes ( Sorokin & Kuenen, 2005), was represented mainly by the *Euhalothece* spp. *Euhalothece* is a single-celled stenohaline cyanobacterium growing optimally at 7% (w/v) NaCl. They depict a morphological variability depending on the concentrations of NaCl and carbonates as well as the pH conditions ( Mikhodyuk *et al.*, 2008). The presence of Euhalothece in Lake Magadi is thus supported by high salts and carbonates ( Table 1). Contrastingly, the results in other soda lakes have shown that *Arthrospira* spp. are the main photosynthetic agents driving primary productivity, with the seasonal occurrence of *Cyanospira*, *Synechococcus,* and *Chroococcus* to augment this process ( Jones *et al.*, 1998). Thirdly, members of the phylum Bacteroidetes were majorly represented by the genera *Rhodothermus*, *Roseinatrobacter*, *Gracilimonas*, *Lewinella*, and *Cytophaga.* Verrumicrobia was represented by *Verrucomicrobium*, *Puniceicoccus*, and *Coraliomargarita.* Bacteroidetes and Verrucomicrobia are known to thrive in high-nutrient environments where they play a role in the degradation of biopolymers such as cellulose and chitin ( Newton *et al.*, 2011). Interestingly, the presence of Bacteroidetes and Verrucomicrobia was often associated with the availability of Cyanobacteria across the sites and the sampling periods ( *Extended data,* Supplementary Table 1 ( Kipnyargis *et al*., 2023a)). Photosynthetic stages of cyanobacteria lead to a high rate of CO _2_ and HCO _3_^-^ consumption, and a consequent increase in pH ( Almeida *et al*., 2011). As a result, nutrient release is enhanced from the sediments of the lake, hence acting as substrates for Bacteroidetes and Verrucomicrobia. Phylum Firmicutes (2.7% of all the reads) was represented by members of Class Clostridium ( *Clostridium*, *Halanaerobium*, *Natranaerobius*, and *Moorella* sp.) and Bacilli ( *Alkalibacterium* and *Anoxybacillus* sp.).

The archaeal community of the Lake Magadi microbiome was represented by three phyla, the Euryachaeota (25% of all the reads), Crenarchaeota (0.01%), and Thaumarchaeota (0.01%). Generally, archaea were more abundant in brine samples with Br1_June and Br1_Sept accounting for 24.5% of total archaea. Previous studies have indicated that archaea are more adapted to saline environments than bacteria ( Mani *et al.*, 2020). Euryachaeota was the most abundant phylum across the sites and the sampling seasons with 87% of all archaeal communities. Euryachaeota has well-adapted inhabitants of hypersaline environments where they play a critical role in ecosystem services such as carbon cycling by functioning as methanogens ( Jiang *et al.*, 2007; Vavourakis *et al.*, 2016). Grant *et al.* (1999) first characterized this phylum in the alkaline saltern of Lake Magadi. The second most abundant group was Thaumarchaeota also known as ammonia-oxidizing agents ( Andreote *et al.*, 2018). In marine environments, their distribution along a salinity gradient has been linked to changes in location, salinity, and sediment depth ( Webster *et al.*, 2015). A one-time sampling of the Lake Magadi hot springs depicted a similar picture with Euryachaeota accounting for up to 28% abundance while Thaumarchaeota and Crenarchaeota were each 1% abundant ( Kambura *et al.*, 2016). Studies in other soda lakes that have detected members of the phylum Euryachaeota and Crenarchaeota ( Ghori *et al.*, 2021), Crenarchaeota, Euryarchaeota, Woesearchaeota, and Pacearchaeota ( Wang *et al.*, 2022). However, explorations in Soda Lake in Salina Preta, Brazil, revealed the contrary where members of Thaumarchaeota were most abundant while Euryarchaeota was least abundant ( Andreote *et al.*, 2018). The most abundant archaeal genera belonged to *Halorubrum* (18.3%), *Salinarchaeum* (5.4%), and *Haloterrigena* (1.3%). Most of these microbes have their habitats in soda lakes and neutral saline environments ( Feng *et al.*, 2005; Mwatha & Grant, 2016; Minegishi *et al.*, 2017; Zhao *et al.*, 2020). Other haloalkaliphilic archaea related to genera *Natronomonas*, *Natrialba*, *Natrococcus*, *Natronobacterium*, *Natronolimnobius*, and *Halorubrum*, all of whom were detected in this study, have previously been isolated from brines of East African soda lakes and Inner Mongolian lakes where salinity values reach > 30%, and pH values of >10 ( Grant and Sorokin, 2011). Overall, the results of this study reflect bacterial composition in many soda lakes around the world ( Sorokin *et al.*, 2014a; Kambura *et al.*, 2016; Mani *et al.*, 2020; Poyraz & Mutlu, 2020; Wang *et al.*, 2022).

Co-occurrence network analysis demonstrates the interactions between microbial taxa, which can be symbiotic or competitive ( He *et al.*, 2019). At the family level revealed the presence of heterogenous microbial communities that co-occur in different sampling sites along the lake as well as others that were unique to a particular site, suggesting mutual interactions of these communities across the sites. For instance, Desulfobacteriaceae were unique to S2. Correspondingly, S2 had the highest concentration of both sulfur and sulfate ions ( Table 1). This family, particularly Desulfonatronum, Desulfonatronospira, Desulfonatronovibrio, and Desulfohalophilus have been shown to thrive in anoxic parts of soda lakes acting as sulfate-reducing bacteria (SRB) through the oxidation of hydrogen and formate or direct disproportionation of sulfite of thiosulfate ( Sorokin *et al.*, 2011). Noteworthy, *Thioalkalivibrio* sp. was not significantly influenced by the physicochemical properties investigated. This suggests that these strains have devised adaptive mechanisms to thrive under the prevailing harsh conditions of Lake Magadi. Indeed, *Thioalkalivibrio* sp., which are sulfur-oxidizing bacteria (SOB), have been found to adapt well in soda lake ecosystems ( Li *et al.*, 2022). Unique to the S6 site were the members of the family Puniceicoccaceae which have also been described in four soda lakes of the Cariboo Plateau in Canada ( Zorz *et al.*, 2019). Cyclobacteriaceae retrieved from the S1 site have established habitats in diverse ecosystems like cold marine regions like algal/microbial mats, haloalkaline soda lakes, Antarctica, freshwater bodies, marine waters, marine sediments, mangroves, hot springs, and mud volcanoes ( Rosenberg *et al.*, 2014). Members of families Rhodobacteraceae and Cyclobacteriaceae have sulfate–oxidizing properties, whereas Burkholderiaceae (unique to S1) have adapted to different ecological niches and are involved in processes such as catabolism of aromatic compounds as well as nitrogen fixation ( Pérez-Pantoja *et al.*, 2012).

Alpha diversity studies revealed that samples in the open waters, particularly S5, had the highest species richness and diversity. However, open waters samples from S2–S4 depicted varying degrees of microbial diversity. While this could not be conclusively explained, it has been suggested that water waves affect the distribution and degradation of organic matter degradation and nutrient cycling, thereby influencing the composition diversity of microbes in water ecosystems ( Zhu *et al.*, 2018). Brine samples (BR1) in June and September had relatively high diversity and evenness indices. Despite high salinity and alkalinity in soda lake brine, the presence of high light intensity and dissolved CO _2_ promotes the growth of photosynthetic microorganisms. Subsequently, these phototrophs generate large quantities of dissolved organic matter (DOM) which become substrates for sustaining the diverse microbial communities ( Banda *et al.*, 2020). Moreover, hot spring samples (S1) exhibited high Shannon and Simpson diversity indices, an indication of high microbial diversity. Research on Soda Lake hot springs revealed a highly active and diverse microbial community, suggesting the high plasticity of these organisms toward extreme environments ( Dadheech *et al.*, 2013; Kambura *et al.*, 2016).

The principal component analysis revealed that compared with other samples, hot spring (S1) and brine (BR1) samples clustered according to their sites (cluster I and II, respectively) ( Figure 3) . This indicates that hot spring and brine samples had distinct community similarities ( Tao *et al.*, 2019). However, samples appeared to cluster based on the changes in the salinity and alkalinity of the sampling sites from low to high salinity and alkalinity clusters. This is an indication that the impact of time point sampling on the community structure was limited. Previous studies have established that salinity is the primary selective force driving the distribution of beta diversity, whereas alkalinity influences microbial richness ( Antony *et al.*, 2013; Boros & Kolpakova, 2018; Banda *et al.*, 2020). Moreover, extreme salinity and alkalinity confine the microbial communities to a few taxa highly adapted to the prevailing conditions ( Oren, 2011).

In terms of water chemistry ( Figure 7; *Extended data*, Supplementary Figure 3 ( Kipnyargis *et al*., 2023a)), pH, temperature, PO _4_^3-^, K, and NO _3_^−^, NH _4_^+^, Mn, Na ^+^, SO _4_^2-^, and TDS influenced the variation of microbial community composition in Lake Magadi. Salinity and alkalinity tend to influence the distribution of the microbial communities in the Soda Lake ecosystem. Specifically, members of genera *Nocardiodes*, *Rhodothermus*, *Haloterrigena*, *Methanomasiliicoccus*, *Halorubrum*, *Palaeococcus*, *Nocardioides*, *Salinarcheum*, *Salinibacter,* and *Euhalothece* had a wide range of physicochemical adaptability. Conversely, *Synechococcus*, *Thioalkalivibrio*, *Cyanobacterium* spp., *Rhodovulum*, *Lewinella*, *Idiomarina*, *Pseudidiomarina*, *Chelatococcus*, *Aliidiomarina*, and *Alkalimonas* were least influenced by the tested physicochemical factors ( *Extended data*, Supplementary Figure 3 ( Kipnyargis *et al*., 2023a)). The archaeal genera *Salinarchaeum* and *Halorubrum Halobellus*, *Halolamina*, *Methanobrevibacter*, and *Halorhabdus* have been strongly associated with salinity and factors such as pH, Mg ^2+^, Na ^+^, K ^+^, Ca ^2+^, and SO _4_^2–^ ( Han *et al.*, 2017). Nitrate appears to drive the structure of the members of the genera *Aliidiomarina*, *Idiomarina,* and *Alkalimonas* ( Figure 4, *Extended data* Supplementary Figure 3 ( Kipnyargis *et al*., 2023a)). Many strains of *Alkalimonas* have been isolated from Chahannor (China), Kulunda Steppe (Russia), and Elementaita (Kenya) soda lakes where they play a role in nitrate reduction and formation of H _2_S ( Ma *et al.*, 2004; Vavourakis *et al.*, 2016). However, in this study, sulfur was negatively correlated with *Alkalimonas.* The *Aliidiomarina* and *Idiomarina* belong to the family Idiomarinaceae and have also been described as nitrogen reducers but poor in carbohydrate utilization ( Chiu *et al.*, 2014).

## Conclusion

Studies combining ecological, physiological, and taxonomic aspects have shown the remarkable diversity of halocaliaphiles in numerous saline and alkaline lakes. The current study determined the structure of the microbial communities in different sampling sites and time points in Lake Magadi and explored the influence of physicochemical parameters on the composition and diversity of these microorganisms. Our findings indicated that results revealed that in comparison to archaea, bacteria are much more diverse in this ecosystem. The bacterial community was dominated by the phyla Proteobacteria, Cyanobacteria, Bacteroidetes Actinobacteria, Firmicutes, and Verrumicrobia. On the other hand, archaea were majorly composed of Euryachaeota while Crenarchaeota, and Thaumarchaeota were found in the least abundance across the sampling seasons and sites. Species richness and diversity varied within open waters while samples from brine (Br_1) and hot spring (S1) revealed high diversity and evenness was recorded. Ordination studies indicated that samples clustered based on salinity and alkalinity rather than the sampling season. Water chemistry depicted high concentrations of sodium salts, HCO _3_^-^, SO _4_^2-^, pH values of 9.8–11.5, temperatures of 27–38°C, and low concentrations of Ca ^2+^, Mg ^2+^, and Cu ^2+^. Temperature, pH, P, K, NO _3_^-^, and TDS significantly influenced the microbial community distribution in Lake Magadi. Our research highlights the composition and diversity of microbial communities across four successive dry months of soda Lake Magadi and the key physicochemical factors influencing them. We recommend future research focus on the functional profiles of samples during and after cyanobacterial blooms, including vertical profile stratification of Lake Magadi. The inclusion of sediment samples will also elucidate the taxonomic and functional profile of anoxic microbial communities.

## Data Availability

NCBI BioProject: Prokaryotic diversity within the hypersaline Lake Magadi in Kenya. Accession number: PRJNA962270. https://www.ncbi.nlm.nih.gov/bioproject/PRJNA962270/ ( Kipnyargis *et al*., 2023b). Figshare: Structure and composition of the microbial communities in hypersaline Lake Magadi: Additional Materials. https://doi.org/10.6084/m9.figshare.22699456 ( Kipnyargis *et al*., 2023a). This project contains the following extended data:
• Supplementary Figure 1.png (The distribution of shared operational taxonomic units (OTUs) based on the month of sampling in Lake Magadi.) • Supplementary Figure 2.png (Percentage read abundance of the top 20 species of the microbial communities collected from Lake Magadi.) • Supplementary Figure 3.png (The influence of physicochemical parameters on the structure of microbial communities in Lake Magadi.) • Supplementary Table 1.xlsx (Percentage abundance of bacterial and archaeal communities in Lake Magadi across the sampling months, broken down by phylum.) • Supplementary Table 2.docx (Mantel test results of the effects of physicochemical factors on microbial structure and composition in Lake Magadi.) Data are available under the terms of the Creative Commons Attribution 4.0 International license (CC-BY 4.0).
