# Supplementary material for: Spatiotemporal structure and composition of the microbial communities in hypersaline Lake Magadi, Kenya
Source: F1000Res. 2024 May 17;13:11. Originally published 2024 Jan 3. [Version 2] doi: 10.12688/f1000research.134465.2 (PMC11555362; doi:10.12688/f1000research.134465.2)
Supplement: Peer review history

# Reviewer report: Reviewer response for version 2

**DOI:** 10.5256/f1000research.166004.r330095

**Published:** 2024-11-11

**Authors:** Addis Simachew

**Affiliations:** 1 Institute of Biotechnology Associate Professor of Microbial Ecology and Biotechnology, Addis Ababa University, Addis Ababa, Ethiopia

I would like to thank the authors for conducting this nice work which fills a gap by assessing microbial communities structure in response to seasonality in Lake Magadi, Kenya, one of the soda lakes in the East African Rift Valley System.

General comments

 The authors have attempted to assess the structure and composition of microbial communities in response to the seasonality and physicochemical parameters of Lake Magadi. Accordingly, the authors assessed the microbial communities structure and composition using three types of samples (hot spring water, open lake water and brine) of selected sampling sites in lake Magadi; sampled at four consecutive months (June to September of 2018). The key research question as pointed out by the authors was “how microbial diversity changes with the fluctuating physicochemical conditions with seasons, specifically wet/rainy and dry seasons?

 In general, the manuscript needs major revision for acceptance due to the following major issues:

 1.The sampling months were successive dry months, as described by the authors, it contradicts with the objective and hypothesis of the study. What does the authors mean by seasonality considering only dry months of the year?.  The study was designed as stated by the authors, to investigate the effect of seasonality, i.e., the effect of dry and wet/rainy seasons on the microbial communities structure and composition. However, the study design did not include the rainy season of the area to answer the research question and test the research hypothesis.  To this end, in the authors concluded that “our research highlights the composition and diversity of microbial communities across four successive dry months of soda Lake Magadi and the key physicochemical factors influencing them” in contradiction to the research objective and hypothesis. 

 2.The number of samples taken from each sample type were not proportional. Only a single   composite sample was taken from the hot spring (S1) and brine (Br1), while five composite samples (S2-S6) were taken from the open lake water. The authors did not justify why such sampling scheme was employed. Unless justified the representativeness and the reliability of the data is questionable.  

 3.Three sample sites (S2, S3 and S4) were on the northern tip of the lake while the other two sample sites (S4 and S5) were on the southern tip of the lake (Figure 1). These sample sites were also around the shore of the lake while the large portion of the lake in the middle was not covered. The key question is that, are the sample sites representative to assess the lake microbial community? What was/were the reason/s to consider such sampling scheme? 

 4.The data for Br1-June, Br1-August, S1-June, S1-August and S2-August are not available. Then, how comparison is possible without having the full set of data for each sample site?  

 5.The temperature of the sample sites were in the range of 27 ^o^C (S1-June and S4-July) to 38.7 ^o^C (S3-June) while the maximum temperature the sample site considered as hot spring was 35.6 ^o^C both in June and September which is less than to some of the other sample sites. What is/are the reason/s for considering the sampling site with low temperature value as hot spring?  

 6.There is a new bacterial classification system. In this new classification system, some phylum names are changed, Proteobacteria to Pseudomonata; Firmicutes to Baccilota etc. Hence, it would be better if the new classification systems is used.

 Specific comments

 Taxonomic composition and structure

- Paragraph 1

1.The proportion of bacteria to archaea varied by season and sampling month (Figure 4)…

 The sampling month  represents the season. Then what do you mean by season and sampling month?

 2. In hot spring water (S1), archaea abundance was the lowest while bacterial abundance was the highest

 3.What was the proportion of archaeal abundance in these months? It would be clear if the percentage values are presented. What about for July and August?(sites 1, 4, and 5)

 4.No such description of sites either in the method nor the result. Please make it consistent for the sake of understanding.

 5. From June to September 2018, bacterial abundance decreased while archaeal abundance increased (Figure 4A)

 6.It would be more informative if the proportion is described in percentage or in numerical.

 The data in Figure 4A shows the microbial abundance at domain level along sampling sites. It would be more informative if the data were presented along months for a specific month. It is very difficult to conclude if the variations was due to month or site specific variations. Moreover, the data for some sampling sites is not complete. For instance, No data for S1 for July, August and the same is true for Br1.

- Paragraph 3

7.S1 and S6 had the least archaeal abundance with 0.08 and 0.8%, respectively. In terms of the sampling month, September had the highest archaeal abundance with 53% followed by August (23%), June (12.7%), and July (11.4%)…

 8.It would be appropriate if this information is included in paragraph 1.

- Paragraph 4

9.The word “sampled” is used several times. It is better if other better word is used.

 10.. (Kipnyargis et al., 2023a))…Why this citation?

- Paragraph 5

11. Halobacteroidaceae, Spirochaetaceae, halanaerobiaceae, and desulfohalobiaceae…

 halanaerobiaceae, and desulfohalobiaceae: start with capital letters

- Paragraph 5

12.Generally, members of genera Nocardiodes, Rhodothermus, Haloterrigena, Methanomasiliicoccus, Halorubrum, Palaeococcus, Nocardioides, Salinarcheum, Salinibacter, and Euhalothece spp. had a wide range of adaptability---

 13.Conversely, Synechococcus, Thioalkalivibrio, Cyanobacterium, Rhodovulum, Lewinella, Idiomarina, Pseudidiomarina, Chelatococcus, Aliidiomarina, and Alkalimonas spp. were least influenced by the tested physicochemical factors---The above statements are contradictory. 

 14.The first statement describes that members of the genera mentioned had a wide range of adaptability which implies that they are less influenced by the physicochemical parameters of the environments. The second statement also indicates the same for members of the microbial genera listed out. It needs attention of the authors.

 …Mn and CO32-. pH and NH4+ …

  The dot next to should be corrected

 …. correlated with Mg2+, Mn, and NO3- …

 Mn should be written in the ionic form as the other species

 …On the other hand, Mn, temperature CO32-, and NH4+ …

 The same comment as above

Is the work clearly and accurately presented and does it cite the current literature?

Yes

If applicable, is the statistical analysis and its interpretation appropriate?

I cannot comment. A qualified statistician is required.

Are all the source data underlying the results available to ensure full reproducibility?

Partly

Is the study design appropriate and is the work technically sound?

Partly

Are the conclusions drawn adequately supported by the results?

Partly

Are sufficient details of methods and analysis provided to allow replication by others?

Partly

Reviewer Expertise:

Haloalkaliphilic Microbial Ecology and Biotechnology

I confirm that I have read this submission and believe that I have an appropriate level of expertise to confirm that it is of an acceptable scientific standard, however I have significant reservations, as outlined above.

# Reviewer report: Reviewer response for version 2

**DOI:** 10.5256/f1000research.166004.r330100

**Published:** 2024-10-23

**Authors:** Cristiano Pedroso-Roussado

**Affiliations:** 1 Universidade de Lisboa Instituto Superior Tecnico, Lisboa, Portugal

I want to thank the authors for performing this work which represents a relevant microbial profile study for bacteria and archaea in Lake Magadi, Kenya. 

 General:

 The work needs a whole revision for refine its text, and allow a more fluid narrative. The methods' section does not match with the results section. The discussion needs to have a more critical take on the findings. Key words does not reveal a clear picture of the work. Hence, I believe this work needs another round of revision before being fully accepted.

 In detail:

 INTRO

 - Alpha-diversity is an index that speaks about diversity within samples and this seems not to be fully understood by the authors. The same for beta-diversity, that speaks about diversity between samples. A more critical take on these indexes is necessary.

 . It is not clear the relevance of measuring TDS.

 . Annotations are not correct: replace "ml" for "mL" and give a space for "X ºC".

 - Is there any reference for the taxonomical update of *Pelagirhabdus fermentum?*

 - What is a lake lagoon?

 - What is the relevance of the isolates that Edwin et al (2019) and Orwa et al (2020) collected with the work you here introduce?

 METHODS

 - It is not clear how many water samples were collected nor at which depth. More details on the methods' section is necessary.

 - Why do you justify the collection of 50 mL per sample instead of >5 L?

 - How long did it take for traveling with samples? And at what temperature?

 RESULTS

 - You should not state the objective of the study in the results' section.

 - Table 2 is showing all the results which is not a conventional way to show different variables (taxonomy vs diversity). I would prefer to see the sequencing QC as well.

 . Does the figure 2 show the three different collection points combined (spring, brine, open waters)? It is needed more clarity for the space and time importance on the results.

 - What does the proportion of bacteria to archaea tell us?

 - It is not mentioned how did you perform the co-occurrence and redundancy analyses.

 - Your study relates to the microbiota and not to the microbiome (Berg et al., (2020)(Ref-1))

Is the work clearly and accurately presented and does it cite the current literature?

Partly

If applicable, is the statistical analysis and its interpretation appropriate?

Partly

Are all the source data underlying the results available to ensure full reproducibility?

Yes

Is the study design appropriate and is the work technically sound?

No

Are the conclusions drawn adequately supported by the results?

Partly

Are sufficient details of methods and analysis provided to allow replication by others?

No

Reviewer Expertise:

Applied microbiology, microbiome analysis, next-generation sequencing, third-generation sequencing.

I confirm that I have read this submission and believe that I have an appropriate level of expertise to confirm that it is of an acceptable scientific standard, however I have significant reservations, as outlined above.

## References

[1] : Microbiome definition re-visited: old concepts and new challenges. *Microbiome* .2020;8(1) : 10.1186/s40168-020-00875-0 10.1186/s40168-020-00875-0 32605663 PMC7329523

# Reviewer report: Reviewer response for version 2

**DOI:** 10.5256/f1000research.166004.r279828

**Published:** 2024-06-17

**Authors:** Marianne Haines

**Affiliations:** 1 University of Calgary, Calgary, Alberta, Canada

Well done getting this all updated. The manuscript is in much better shape. I can see all the hard work you have put into it. Well done. There are a few minor things I would suggest amending and then that’s all from me. Good luck with all your future science – I hope it brings you much joy and remains inspiring. See below:

**Abstract**

 Abstract: background

- “However, it is not yet clear if the change in physiochemical changes influences the spatiotemporal diversity and structure of microbial communities in these ecosystems.” VS “The distribution and diversity of microbial communities in hypersaline lakes are mainly affected by physicochemical parameters (Tazi et al., 2014)” in the 2 ^nd^ paragraph of the Introduction. These two statements are contradictory. Perhaps you are trying to say that the influence of physiochemical parameters *within a single lake* have not been explored.

Abstract: methods

- You have repeated a sentence twice

Abstract: results

- The “composition” of Archaea cannot be higher. I think you mean the total abundance of Archaea was higher than that of Bacteria.
- What is P+ ? Phosphorous? Why does it have a “+”?

**Methods:**

- All these updates make this much better

**Results**

- I still think Table 2 needs to define what an “observed species” is. Please refer to review 1 for more on this. Is it the number of unique species that were assigned?
- “Low alkalinity and salinity samples (pH 9.8 – 10.5; 10,300 ppm – 70,500 ppm) formed cluster I with nine samples (S1_06, S1_09, S5_06, S5_09, S5_08, S5_07, S6_08, S6_06, and S6_07). Moderately alkaline and saline samples (pH 10.5 – 10.6; **63**, 900 ppm – 100,000 ppm)” – just need to remove the spacing between 63, and 900 to reader doesn’t get confused and think these are two separate numbers. My interpretation is that this is actually 63,900 ppm?
- Still need to be more specific with this one “pH and NH4 ^+^ appear to positively correlate with the structure of the members of the genus *Salinarcheum* (R ^2^ = 0.245; p < 0.004), but negatively correlated with NO _3_^-^.” How does something positively correlate with the “structure of the members” – do you mean at increasing pH and ammonium concentrations more members of that genus were present? The subject of this sentence is “pH and NH4+” so as it is this sentence implies that pH and NH4+ were negatively correlated the nitrate. I don’t think that is what you mean, I think you mean that with increases in NO3- the presence and/or abundance of *Salinarcheum* was reduced. Is that it?

**Discussion**

- This has come a long way, I can really see that you have now compared your results to others. Well done.

**Conclusion**

- “halocaliaphiles” spelling
- Much better conclusion

Is the work clearly and accurately presented and does it cite the current literature?

Partly

If applicable, is the statistical analysis and its interpretation appropriate?

I cannot comment. A qualified statistician is required.

Are all the source data underlying the results available to ensure full reproducibility?

Yes

Is the study design appropriate and is the work technically sound?

Yes

Are the conclusions drawn adequately supported by the results?

Partly

Are sufficient details of methods and analysis provided to allow replication by others?

Yes

Reviewer Expertise:

alkaline soda lake communities, alkaliphilic cyanobacteria

I confirm that I have read this submission and believe that I have an appropriate level of expertise to confirm that it is of an acceptable scientific standard.

# Reviewer report: Reviewer response for version 1

**DOI:** 10.5256/f1000research.147523.r251335

**Published:** 2024-05-11

**Authors:** Srijak Bhatnagar

**Affiliations:** 1 Athabasca University, Athabasca, Alberta, Canada

**Major Feedback:**

1. Please run us statistical test on the alpha diversity values as it appears the August values could be very similar to June values statistical test is required to ensure the difference mentioned by the authors
2. Sequence analysis subsection under result, line 2: average read length is listed as 525bp The Amplicon expected from the primer mentioned should be around 300 and the length of my seek is also around 300bp. How is read length possible?
3. In various places site, S1 is described as a hot spring, but the temperature is shown as roughly around 35°C. How is this a hot spring?
4. Subsection taxonomic, composition, and structure: “Population increased due to evaporative iron concentration”. This is a causal statement, and no evidence of causation is shown in the study.
5. Figure 4B: the results mention Euryarcheota as the most abundant phylum. This is not evident from the figure. Either the legend is wrong, or the result is misinterpreted
6. Subsection physicochemical drivers: “TDS has a positive influence”. This is a subjective statement with no evidence provided. Unsure what positive influence here means.
7. Use of homonyms: Certain terms have a well-defined and established meaning and could cause confusion, please avoid them. For eg.,
  -  Figure 6:” exclusive site of isolation”. Isolation is a specific microbial technique, which was not employed in this study.
  - DOM -> Dissolved Organic Matter, but in the manuscript used for Dead Organic Materials
8. The authors missed the opportunity to guide the discussion by focusing on the temporal changes in physicochemical factors driving the microbial community changes and then discussing possible reasons. The way the discussion is currently framed, it is a mix of results retelling and describing various taxa that were observed by other publications. 

**Minor Issues:**

- There is no mention of Phix filtering and sequence processing under methods
- Typo in paragraph two of microbial community analysis, subsection Bray Curtis dissimilarity, written as Bray dissimilarity
- Table 1: add the charge to the measured ions
- Results first paragraph line 3: various ions and cations-> various ions or various anions and cations
- Please mention the medium library size. It is more informative than the average size.
- Incomplete sentence. beta diversity studies, subsection, line 2: samples better individual community similarity
- Figure 3: Label the clusters as described in the results section
- Figure 5: plastering of samples and taxa that here would be helpful and visualising the heat map while it’s understandable that samples are shown by month class based upon composition, should be able to substantiate some of the observations made by the authors.
- Discussion: “The concentration of these elements” – >“The concentration of these ions”

Is the work clearly and accurately presented and does it cite the current literature?

Partly

If applicable, is the statistical analysis and its interpretation appropriate?

Partly

Are all the source data underlying the results available to ensure full reproducibility?

Yes

Is the study design appropriate and is the work technically sound?

Yes

Are the conclusions drawn adequately supported by the results?

Partly

Are sufficient details of methods and analysis provided to allow replication by others?

Yes

Reviewer Expertise:

Bioinformatics, microbiomes, and environmental microbiology

I confirm that I have read this submission and believe that I have an appropriate level of expertise to state that I do not consider it to be of an acceptable scientific standard, for reasons outlined above.

# Reviewer report: Reviewer response for version 1

**DOI:** 10.5256/f1000research.147523.r251341

**Published:** 2024-04-01

**Authors:** Marianne Haines

**Affiliations:** 1 University of Calgary, Calgary, Alberta, Canada

**Summary**

 The authors sampled 7 sites representing spring, brine, and open waters in Lake Magadi, Kenya, over a 4-month period. Each sample underwent physiochemical testing and DNA extraction for amplicon sequencing and subsequent microbial community analysis. The authors thoroughly explore the composition of the microbial communities at each site and timepoint. Community composition is explored through standard taxon abundance measures and multiple diversity measures. Physiochemical drivers of the success (abundance) of different taxa are also evaluated. This work is valuable to microbial ecologists interested in extreme environments, notably, alkaline soda lakes.

**Comments**

 Firstly, thank-you so much for your commitment to science. Well done! It takes a lot of work to get a manuscript to this stage. You have put together a very comprehensive analysis of your community data. Your results are stated very well and are nicely presented in figures. I really enjoyed the in-depth exploration of the community data. Overall, the manuscript is in good shape, although there are some places, particularly in the discussion and conclusion, where I think it requires some work.

**Abstract**

- Add in that you did 16S *AND *analysed physiochemical properties in the methods 

- “Furthermore, salinity and alkalinity affect β-diversity rather than the sampling site or seasonality.” Probably seasonality and site also impact diversity but they are masked by the stronger drivers of salinity and alkalinity. You could rephrase this to say salinity and alkalinity had a *stronger* or the *most notable* impact on beta diversity. Indeed you actually directly contradict this point in the last sentence: “Multivariate analysis revealed significant spatial and temporal effects on β-diversity and salinity and alkalinity were the major drivers of microbial composition in Lake Magadi.”

- “The effects of physicochemical parameters on the microbial community structure showed that temperature, pH, P+, K+, NO3 -, and total dissolved solids (TDS) had a positive correlation with the microbial community structure” I don’t know what you mean by positive correlation on microbial community structure. Explain.

**Introduction**

- What do you mean by “validly described”, be more specific what your definition of valid descriptions are.

- This is stated really nicely, “A key ecological question is how microbial diversity changes with the fluctuating physicochemical conditions with seasons.”

- “In this study, we explored the spatiotemporal variation in the microbial community AND physiochemical properties of the water over four months at different sites in Lake Magadi using Illumina 16S sequencing.” 16S is more specific than the type of sequencing.

**Methods**

- The naming of samples makes it easy to distinguish brine from the other sample types but it is hard to know that S1 is the only spring.

- Figure 1 – it is hard to see which sites are S3 and S4

- Bit unnecessary to have the type of cool box.

- I haven’t done extensive diversity analysis of 16S data so I can’t comment on these methods.

- Should include some text that states that the primer is designed to capture both Bacteria and Archaea.
- It would be helpful to state early on in the piece which seasons these months fall in to in Kenya. Are these warm or cool months, rainy or dry? Not all readers will know this immediately.

**Results**

 There was an uneven distribution in sampling times, that needs to be mentioned. Why did this happen? How could it have influenced/biased your results?

**Physicochemical properties of the sampling sites**

- Table 1 needs units beside each metric in column 1 even though I see they are stated in the body of the text, one should be able to look at the Table without needing to seek clarification from the text.

- Why are there months missing for some of the samples? If data wasn’t collected for those months, how did that impact RDA which was used to test the correlation between physiochemical parameters and genera? Why these samples are missing and how the analysis overcame this needs to explained.

**Sequence analysis and diversity studies**

- Table 2: What are “observed species”, you say that taxa were assigned to genus level, not to species level. Is this column actually talking about the number of different genera? This column should have the 00s removed.

- Table 2 rows should be ordered by sample and then chronologically.

**Alpha diversity studies**

- This is the first time in the piece you have mentioned the spring is a hot spring. That needs to be updated in the methods.

- Figure 2 has the x axis categories in alphabetical order but I think from the perspective of the reader this should be presented chronologically. Seasonality usually has a profound impact on community structure, it would be nice to visualise easily if there are (or are not) diversity trends based on seasonality.

- In Figure 2 legend, you state “Statistical significance was determined at p < 0.05” but I don’t see any indication of statistical significance conveyed on the graph so I don’t think this sentence is necessary. 

- I like that you have presented the results from so many different measures of species diversity here. It is not necessary to do it for this work, but one way to simplify this type of graph (especially because all tests show similar trends) would be to normalise all values and then plot them on a single graph with box plot colours indicating the test type. At present, your coloured key here is unnecessary because you already have the months as categories in the x-axis.

- “The alpha diversity indices showed that high microbial diversity was recorded in the month of August, followed by September, June, and July in that order (Figure 2).” I don’t think these results clearly show this, they are too variable and overlapping.

**Beta diversity studies**

- “Low alkalinity and salinity samples (pH 9.8 – 10.5; 10 ***what is this number?***, 300 ppm – 70,500 ppm).” Same thing with the 63 in the medium cluster, and then that number is missing in the high cluster.

- Figure 3 needs to tell me which clusters are which, preferably on top of the graph are in the legend. That’s cool that they cluster like that.

**Taxonomic composition and structure**

- “The results indicate that the archaeal population increased due to evaporative ion concentration while bacteria abundance was higher where the ion concentration was lower (sites 1, 4, and 5) (Table 1).” I don’t think you can conclude this from your results. You might say there is a trend and it could be because of ion concentration. This is a discussion point and shouldn’t be in the results section.

- Figure 4: I am a bit confused with the alignment of different samples, why are there gaps between bars in some cases but not in others? I think 4A and 4B should have their samples aligned vertically according to month and sample site. If there are gaps where one sample was completed for Bacteria and not for Archaea that’s fine, but the figure needs to simply explain why. Gaps between sample types makes in 4A but things go astray in 4B.

- Is the relative abundance on the y-axis of figure 4A based on read number? How was that calculated?
- Figure 6: What a cool plot. This is a cool way to visualise data. I like it!  
- Figure 7: In the legend, “Samples corresponding to their sampling month are indicated as indicated”. This is a bit of a clumsy sentence. Good news is the key is completely self-explanatory so you just delete it. You don’t need to explain TDS again, its common enough in the field and you already have described it in the methods.  
- “Notably, members of the phylum Proteobacteria were the most dominant group across all the sampling sites, accounting for 35% abundance.”  Is this abundance based on all sample site and timepoints pooled together? That should be clarified.
- “Idiomarina vacuolatum was sampled across all the sampling seasons but varied in structure across the sampling sites.” Do you mean varied in abundance? Be as specific as you can when stating results.

**Physicochemical drivers of bacterial and archaeal community structure**

- “Overall, temperature, pH, P+, K+, NO3-, and TDS had a positive influence on the microbial community structure.” – what does this mean? What do you define as a positive influence?

- “were adapted to fewer physiochemical factors” bold statement. Keep it more objective here? You could say that these species were not found in high concentrations of X, Y, Z.

- “pH appears to positively correlate with the structure of the members of the genus Salinarcheum”, what does structure of members mean?

**Discussion**

 Where this manuscript needs attention is in the discussion. In general, discussion **clarity and reasoning **needs improvement. This is particularly evident when citing other literature. There were a few places I wasn’t sure what the authors meant and there are some repeated / missing words.

- “Furthermore, sulfate concentration (39–958 ppm) was lower than that of lakes Sidi Ameur and Himalatt (Algeria) (Boutaiba et al., 2012).” This isn’t a meaningful reference to use here. I don’t understand why this single comparison (of 2 lakes in Algeria) is important to the discussion. Sulfate concentration varies quite a lot throughout soda lakes. Is this sulfate concentration low or high relative to soda lakes in general?

- “The concentrations of these elements (except pH) were varied across the sampling months and sites, suggesting that the lake chemistry is constantly changing in its constituent elements” This isn’t a suggestion … you measured it. Instead you could say “measured elements were variable from site to site and fluctuated with time.

- “Hypersaline lakes are characterized by …” The remainder of this paragraph is not relevant to the discussion. It is either introduction material or should be deleted. It does not “discuss” your results.

- “Under the extremes of salinity and alkalinity, microorganisms in soda …” This entire paragraph is also introduction material or should be deleted. It does not discuss the results. 

- “Notably, the phylum Proteobacteria was the most dominant group across all the sampling sites, accounting for 35% abundance.” Be specific about what the abundance means, it is 35% of all reads from all timepoints and samples sites were attributed to Proteobacteria?

- “They were followed by the phylum Cyanobacteria (14.2%) represented mainly by the *Euhalothece* spp. Euhalothece is a single- celled stenohaline cyanobacterium that grow optimally at 7% (w/v) NaCl.” This is where you can compare to the salt concentration in your system – is it above or below this?

- It seems that Eulohaleaceae are the main bacterial phototrophs in your system. So the following discussion about Spirulina doesn’t make too much sense. You could say this is *in contrast *to many other soda lakes systems where Arthrospira dominates. Does Lake Magadi have more NaCl than other lakes, is that why Eulohaleaceae and not Arthrospira dominate? Have either of these two taxa been found to be common in the lake in other studies?

- “Our findings showed a shift in bacterial composition throughout the sampling seasons where a high abundance of Actinobacteria and Proteobacteria was accompanied by a lower abundance of Actinobacteria.” How is Actinobacteria in high abundance and low abundance?

- “Interestingly, the presence of Bacteroidetes was often associated with the availability of Cyanobacteria across the sampling periods” Keep going here… why could this be the case?

- “However, the abundance of Firmicutes, which possess diverse metabolic capacities and are resistant to oxygen limitations (Martiny et al., 2006) depicted an uneven behavior in relation to Actinobacterial composition.” What do you mean depicted an uneven behaviour?

- “Grant et al. (1999) first characterized this phylum IN the alkaline saltern of Lake Magadi.”

- “Their distribution along a salinity gradient in estuarine sediments may be linked to changes in location and/or salinity as well as gradient sediment depth (Webster et al., 2015).” I don’t understand what you mean here.

- “Crenarchaeota (Ghori et al., 2021), Crenarchaeota, Euryarchaeota, Woesearchaeota, and Pacearchaeota, Euryachaeota and Woesearchaeota (Wang et al., 2022).” Crenarcheaota and Woesearcheota are repeated in this.

- “Remarkably, Thioalkalivibrio sp. was not significantly affected by the physicochemical properties investigated.” Yes! Good observation. But why? Tell me why it is remarkable or unexpected. E.g., “We had expected that Thioalkalivibrio would have responded positively to XYZ…

- “However, open waters samples from S2–S4 depicted varying degrees of community structure and this could be due to variations in the intertidal water zones (Zhu et al., 2018).” Lacking specificity, what do you mean by community structure here, diversity / abundance / species type? What do you mean by variations in intertidal water zones? What type of variations?

- “This phenomenon can be attributed to the maintenance of homogenous abundances by microbial communities during brine formation, hence resulting in higher biodiversity (Banda et al., 2020).” Homogenous abundances of each microbe as evaporation occurs? Why would this happen?

- “Hot spring samples collected in September showed high species diversity indices.” Microbial samples from Lake Magadi hot springs were established to be stable and active (Kambura et al., 2016).” Relevance? Are high species diversity indices unusual in hot springs? Is it unusual to have a stable, active community? What does stable mean here? Similarly structured over time? Is that what is seen at S1? There are only two samples from S1 so that conclusion cannot be drawn anyway.

**Conclusion**

 The conclusion needs some work. The clarity needs to be improved by being much more specific.

- “The results depict a great deal of diversity in bacterial diversity as compared to archaea.” Consider rephrasing this.

- “Salinity and alkalinity are the main drivers of the microbial community in the lake.Overall, members of Nocardiodes, Rhodothermus, Haloterrigena, Methanomasiliicoccus, Halorubrum, Palaeococcus, Nocardioides, Salinarcheum, Salinibacter, and Euhalothece had a wide range of adaptability.” Does this mean these members have a wide range of adaptability to salinity and alkalinity or are present in many sites?

- “Conversely, Synechococcus, Thioalkalivibrio, Cyanobacterium spp., Rhodovulum, Lewinella, Idiomarina, Pseudidiomarina, Chelatococcus, Aliidiomarina, and Alkalimonas were affected by fewer physicochemical factors.” Does this also mean they had a wide range of adaptability? Or does this mean they weren’t positively correlated with particular nutrients?

- “…have a positive correlation with microbial community structure.” What is a positive correlation with microbial community structure? How do they influence the community structure?

Is the work clearly and accurately presented and does it cite the current literature?

Partly

If applicable, is the statistical analysis and its interpretation appropriate?

I cannot comment. A qualified statistician is required.

Are all the source data underlying the results available to ensure full reproducibility?

Yes

Is the study design appropriate and is the work technically sound?

Yes

Are the conclusions drawn adequately supported by the results?

Partly

Are sufficient details of methods and analysis provided to allow replication by others?

Yes

Reviewer Expertise:

alkaline soda lake communities, alkaliphilic cyanobacteria

I confirm that I have read this submission and believe that I have an appropriate level of expertise to confirm that it is of an acceptable scientific standard, however I have significant reservations, as outlined above.

# Response

**Published:** 2024-04-30

**Authors:** Alex Kipnyargis

**Affiliations:** Biological Sciences, University of Embu, Embu, Kenya

- Add in that you did 16S *AND *analysed physiochemical properties in the methods 
- **Response: Methods (paraphrased): **Using 16S rRNA gene amplicon sequencing, we investigated the diversity and structure of microbial communities in water and brine samples taken from Lake Magadi between June and September 2018. Physicochemical parameters were also analyzed for every sampling site.

- “Furthermore, salinity and alkalinity affect β-diversity rather than the sampling site or seasonality.” Probably seasonality and site also impact diversity but they are masked by the stronger drivers of salinity and alkalinity. You could rephrase this to say salinity and alkalinity had a *stronger* or the *most notable* impact on beta diversity. Indeed you actually directly contradict this point in the last sentence: “Multivariate analysis revealed significant spatial and temporal effects on β-diversity and salinity and alkalinity were the major drivers of microbial composition in Lake Magadi.”
- **Response: The sentence **“Furthermore, salinity and alkalinity affect β-diversity rather than the sampling site or seasonality.” **is deleted completely.**
**The statement should now reads: **"Multivariate analysis revealed that salinity and alkalinity were the major drivers of changes in the structure of microbial communities in Lake Magadi."
- “The effects of physicochemical parameters on the microbial community structure showed that temperature, pH, P+, K+, NO3 -, and total dissolved solids (TDS) had a positive correlation with the microbial community structure” I don’t know what you mean by positive correlation on microbial community structure. Explain.
- **Response:  The statement now reads **“The findings demonstrated that temperature, pH, P+, K+, NO3 -, and total dissolved solids (TDS) contributed majorly to diversity observed in the microbial community."

**Introduction**

- What do you mean by “validly described”, be more specific what your definition of valid descriptions are.
- **Response: **"validly described" is replaced with "previously described"

- This is stated really nicely, “A key ecological question is how microbial diversity changes with the fluctuating physicochemical conditions with seasons.”
- **Response: **Thank you.

- “In this study, we explored the spatiotemporal variation in the microbial community AND physiochemical properties of the water over four months at different sites in Lake Magadi using Illumina 16S sequencing.” 16S is more specific than the type of sequencing.
- **Response:** "Illumina" **will be deleted. It will now read: **“In this study, we explored the spatiotemporal variation in the microbial community and physiochemical properties of the water over four months at different sites in Lake Magadi using  16S rRNA gene sequencing.”

**Methods**

- The naming of samples makes it easy to distinguish brine from the other sample types but it is hard to know that S1 is the only spring.

**Response:** The sentence after the GPRS coordinates describes the features of the sampling sites

- Figure 1 – it is hard to see which sites are S3 and S4

**Response: **This has been corrected and a new map will be submitted

- Bit unnecessary to have the type of cool box.

**Response:** *This* *has been* *removed*

- I haven’t done extensive diversity analysis of 16S data so I can’t comment on these methods.

**Response:** No response since it is a comment.

- Should include some text that states that the primer is designed to capture both Bacteria and Archaea.

**Response:** That sentence will now read: “The V4 hypervariable region of the 16S rRNA genes was amplified using the universal primers for bacteria and archaea 515F (5′-GTGCCAGCMGCCGCGGTAA-3′) and 806R (5′-GGACTACHVGGGTWTCTAAT-3′)”

  

- It would be helpful to state early on in the piece which seasons these months fall in to in Kenya. Are these warm or cool months, rainy or dry? Not all readers will know this immediately.

**Response: **The dry season lasted for all of the sampling months, with June marking the start of the season and September being the driest month. This has been effected in the manuscript.

**Results**

 There was an uneven distribution in sampling times, that needs to be mentioned. Why did this happen? How could it have influenced/biased your results?

**Response:** You are right. Uneven distribution in sampling sites was a result of:

 1. S1 and Br_1  (hot spring and brine, respectively were only available in June and September).

 2.  The DNA extraction protocol did not yield sufficient DNA for sequencing in sample S2 for August 2018.

**Physicochemical properties of the sampling sites**

- Table 1 needs units beside each metric in column 1 even though I see they are stated in the body of the text, one should be able to look at the Table without needing to seek clarification from the text.

**Response:** This has been done as suggested.

- Why are there months missing for some of the samples? If data wasn’t collected for those months, how did that impact RDA which was used to test the correlation between physiochemical parameters and genera? Why these samples are missing and how the analysis overcame this needs to be explained.

**Response:**

 1. S1 and Br_1  (hot spring and brine, respectively were only available in June and September).

 2.  The DNA extraction protocol did not yield sufficient DNA for sequencing in sample S2 for August 2018.

 However, the Good’s coverage (Table 2) ranged between 0.81–0.96, indicating that the sequencing depth was sufficient to cover most microbial species information and the sample size was sufficient to reflect the diversity differences among different communities.

**Sequence analysis and diversity studies**

- **Table 2:** What are “observed species”, you say that taxa were assigned to genus level, not to species level. Is this column actually talking about the number of different genera? This column should have the 00s removed.

**Response:** The assignment to genus level is a more confident way of taxonomic assignment while using 16S rRNA gene. Qiime would pick out the species identified in the OTUs, but we would not be confident to pick out species to difficulties surrounding 16S in resolving closely related species. We would probably remove that statement and recommend that future studies should consider the use of Amplified Sequence Variants (ASVs) to confidently assign species. The 00s in the “observed species” column have been removed.

- Table 2 rows should be ordered by sample and then chronologically.

**Response:** This has been ordered accordingly.

**Alpha diversity studies**

- This is the first time in the piece you have mentioned the spring is a hot spring. That needs to be updated in the methods.

**Response: **The word hot spring will be used consistently.

- Figure 2 has the x axis categories in alphabetical order but I think from the perspective of the reader this should be presented chronologically. Seasonality usually has a profound impact on community structure, it would be nice to visualise easily if there are (or are not) diversity trends based on seasonality.

**Response: **Figure 2 has been presented in chronological order.

- In Figure 2 legend, you state “Statistical significance was determined at p < 0.05” but I don’t see any indication of statistical significance conveyed on the graph so I don’t think this sentence is necessary. 

**Response: **This has been removed

- I like that you have presented the results from so many different measures of species diversity here. It is not necessary to do it for this work, but one way to simplify this type of graph (especially because all tests show similar trends) would be to normalise all values and then plot them on a single graph with box plot colours indicating the test type. At present, your coloured key here is unnecessary because you already have the months as categories in the x-axis.

**Response:** I have removed the key

- “The alpha diversity indices showed that high microbial diversity was recorded in the month of August, followed by September, June, and July in that order (Figure 2).” I don’t think these results clearly show this, they are too variable and overlapping.

**Response: **You are right, I will rephrase this sentence.

**Beta diversity studies**

- “Low alkalinity and salinity samples (pH 9.8 – 10.5; 10 ***what is this number?***, 300 ppm – 70,500 ppm).” Same thing with the 63 in the medium cluster, and then that number is missing in the high cluster.

**Response:  **The spaces between the numbers have been removed and the numbers read appropriately. Numbers for high cluster have been inserted.

- Figure 3 needs to tell me which clusters are which, preferably on top of the graph are in the legend. That’s cool that they cluster like that.

**Response:  **A brief explanation of the clusters has been inserted in the figure caption.

**Taxonomic composition and structure**

- “The results indicate that the archaeal population increased due to evaporative ion concentration while bacteria abundance was higher where the ion concentration was lower (sites 1, 4, and 5) (Table 1).” I don’t think you can conclude this from your results. You might say there is a trend and it could be because of ion concentration. This is a discussion point and shouldn’t be in the results section.

**Response: **Good suggestion. I have paraphrased the sentence

- Figure 4: I am a bit confused with the alignment of different samples, why are there gaps between bars in some cases but not in others? I think 4A and 4B should have their samples aligned vertically according to the month and sample site. If there are gaps where one sample was completed for Bacteria and not for Archaea that’s fine, but the figure needs to simply explain why. Gaps between sample types makes in 4A but things go astray in 4B.

**Response: **The bars have been ordered according to sample and month.

- Is the relative abundance on the y-axis of figure 4A based on read number? How was that calculated?
- Figure 6: What a cool plot. This is a cool way to visualise data. I like it!

**Response: **Thank you for the comment.

- Figure 7: In the legend, “Samples corresponding to their sampling month are indicated as indicated”. This is a bit of a clumsy sentence. Good news is the key is completely self-explanatory so you just delete it. You don’t need to explain TDS again, its common enough in the field and you already have described it in the methods.

**Response:** This has been corrected

- “Notably, members of the phylum Proteobacteria were the most dominant group across all the sampling sites, accounting for 35% abundance.”  Is this abundance based on all sample site and time points pooled together? That should be clarified.

**Response:** This is a cumulative abundance based on all the time points and sites. The clarification has been effected in the manuscript.

- “Idiomarina vacuolatum was sampled across all the sampling seasons but varied in structure across the sampling sites.” Do you mean varied in abundance? Be as specific as you can when stating results.

**Response:  **Yes, it is the abundance. I have changed this in the manuscript.

**Physicochemical drivers of bacterial and archaeal community structure**

- “Overall, temperature, pH, P+, K+, NO3-, and TDS had a positive influence on the microbial community structure.” – what does this mean? What do you define as a positive influence?

**Response: **You are right. The sentence has been changed to read “The results demonstrated that temperature, CaCO _3_, pH, P ^+^, K ^+^, NO _3_^-^, and TDS influenced the microbial community structure.”

- “were adapted to fewer physiochemical factors” bold statement. Keep it more objective here? You could say that these species were not found in high concentrations of X, Y, Z.

**Response:   **This has been corrected to capture the aspect of objectivity.

- “pH appears to positively correlate with the structure of the members of the genus Salinarcheum”, what does the structure of members mean?

**Response:** Structure means how members of a particular microbial community distribute across seasons and sites.

**Discussion**

 Where this manuscript needs attention is in the discussion. In general, discussion **clarity and reasoning **needs improvement. This is particularly evident when citing other literature. There were a few places I wasn’t sure what the authors meant and there are some repeated / missing words.

- “Furthermore, sulfate concentration (39–958 ppm) was lower than that of lakes Sidi Ameur and Himalatt (Algeria) (Boutaiba et al., 2012).” This isn’t a meaningful reference to use here. I don’t understand why this single comparison (of 2 lakes in Algeria) is important to the discussion. Sulfate concentration varies quite a lot throughout soda lakes. Is this sulfate concentration low or high relative to soda lakes in general?

**Response:** More comparisons have been introduced in the manuscript to capture similar, higher, and lower concentrations of sulfates in soda lakes.

- “The concentrations of these elements (except pH) were varied across the sampling months and sites, suggesting that the lake chemistry is constantly changing in its constituent elements” This isn’t a suggestion … you measured it. Instead you could say “measured elements were variable from site to site and fluctuated with time.

**Response: **Thank you. I have borrowed your words verbatim and the sentence reads well now.

- “Hypersaline lakes are characterized by …” The remainder of this paragraph is not relevant to the discussion. It is either introduction material or should be deleted. It does not “discuss” your results.

**Response: **That part has been paraphrased to explain the reason for high and stable pH. I felt that since pH was relatively stable across seasons and site, it is important to explain how this happens.

- “Under the extremes of salinity and alkalinity, microorganisms in soda …” This entire paragraph is also introduction material or should be deleted. It does not discuss the results. 

**Response: **I agree. This paragraph has been entirely deleted.

- “Notably, the phylum Proteobacteria was the most dominant group across all the sampling sites, accounting for 35% abundance.” Be specific about what the abundance means, it is 35% of all reads from all timepoints and samples sites were attributed to Proteobacteria?

**Response: **Proteobacteria accounted for 35% of all the reads.

- “They were followed by the phylum Cyanobacteria (14.2%) represented mainly by the *Euhalothece* spp. Euhalothece is a single- celled stenohaline cyanobacterium that grow optimally at 7% (w/v) NaCl.” This is where you can compare to the salt concentration in your system – is it above or below this?

**Response: **I have supported this by a sentence reading: **“**The existence of *Euhalothece* in Lake Magadi is thus supported by high salts and carbonates (Table 1).”

- It seems that Eulohaleaceae are the main bacterial phototrophs in your system. So the following discussion about Spirulina doesn’t make too much sense. You could say this is *in contrast *to many other soda lakes systems where Arthrospira dominates. Does Lake Magadi have more NaCl than other lakes, is that why Eulohaleaceae and not Arthrospira dominate? Have either of these two taxa been found to be common in the lake in other studies?

**Response: **The sentence has been paraphrased to capture the contrasting aspect of Lake Magadi with other soda lakes.

- “Our findings showed a shift in bacterial composition throughout the sampling seasons where a high abundance of Actinobacteria and Proteobacteria was accompanied by a lower abundance of Actinobacteria.” How is Actinobacteria in high abundance and low abundance?

**Response: **The entire paragraph does not discuss the results adequately and has been deleted

- “Interestingly, the presence of Bacteroidetes was often associated with the availability of Cyanobacteria across the sampling periods” Keep going here… why could this be the case?

**Response: **The sentence has been enhanced to explain how photosynthetic cyanobacteria contribute towards the generation of substrates for Bacteroidetes and Verrumicrobia.

- “However, the abundance of Firmicutes, which possess diverse metabolic capacities and are resistant to oxygen limitations (Martiny et al., 2006) depicted an uneven behavior in relation to Actinobacterial composition.” What do you mean depicted an uneven behaviour?

**Response: **The entire paragraph does not discuss the results adequately and has been deleted.

- “Grant et al. (1999) first characterized this phylum IN the alkaline saltern of Lake Magadi.”

**Response: **The word “in” has been inserted appropriately. Thank you!

- “Their distribution along a salinity gradient in estuarine sediments may be linked to changes in location and/or salinity as well as gradient sediment depth (Webster et al., 2015).” I don’t understand what you mean here.

**Response: **This sentence has been paraphrased

- “Crenarchaeota (Ghori et al., 2021), Crenarchaeota, Euryarchaeota, Woesearchaeota, and Pacearchaeota, Euryachaeota and Woesearchaeota (Wang et al., 2022).” Crenarcheaota and Woesearcheota are repeated in this.

**Response: **True. This has been deleted

- “Remarkably, Thioalkalivibrio sp. was not significantly affected by the physicochemical properties investigated.” Yes! Good observation. But why? Tell me why it is remarkable or unexpected. E.g., “We had expected that Thioalkalivibrio would have responded positively to XYZ…

**Response:  **This has been changed to explain the reason for the effect.

- “However, open waters samples from S2–S4 depicted varying degrees of community structure and this could be due to variations in the intertidal water zones (Zhu et al., 2018).” Lacking specificity, what do you mean by community structure here, diversity / abundance / species type? What do you mean by variations in intertidal water zones? What type of variations?

**Response: **Structure has been replaced with diversity. The sentence has been paraphrased to capture the aspect of water waves and their effect on the distribution of nutrients. This could explain the variation in the diversity of microbes in water ecosystems.

- “This phenomenon can be attributed to the maintenance of homogenous abundances by microbial communities during brine formation, hence resulting in higher biodiversity (Banda et al., 2020).” Homogenous abundances of each microbe as evaporation occurs? Why would this happen?

**Response: **The sentence does not justify the diversity enough. I have introduced another theorem. “Despite high salinity and alkalinity in soda lake brine, the presence of high light intensity and dissolved CO _2_ promotes the growth of photosynthetic microorganisms.

 Subsequently, these phototrophs generate large quantities of dissolved organic matter (DOM) which become substrates for sustaining the diverse microbial communities (Banda 2020).”

- “Hot spring samples collected in September showed high species diversity indices.” Microbial samples from Lake Magadi hot springs were established to be stable and active (Kambura et al., 2016).” Relevance? Are high species diversity indices unusual in hot springs? Is it unusual to have a stable, active community? What does stable mean here? Similarly structured over time? Is that what is seen at S1? There are only two samples from S1 so that conclusion cannot be drawn anyway.

**Response: **I have rewritten the statement to capture the relationship of our findings with other studies on hot springs and the reason for such high diversity studies. Research on Soda Lake hot springs revealed a highly active and diverse microbial community, suggesting the high plasticity of these organisms toward extreme environments.

**Conclusion**

 The conclusion needs some work. The clarity needs to be improved by being much more specific.

- “The results depict a great deal of diversity in bacterial diversity as compared to archaea.” Consider rephrasing this.

- “Salinity and alkalinity are the main drivers of the microbial community in the lake. Overall, members of Nocardiodes, Rhodothermus, Haloterrigena, Methanomasiliicoccus, Halorubrum, Palaeococcus, Nocardioides, Salinarcheum, Salinibacter, and Euhalothece had a wide range of adaptability.” Does this mean these members have a wide range of adaptability to salinity and alkalinity or are present in many sites?

- “Conversely, Synechococcus, Thioalkalivibrio, Cyanobacterium spp., Rhodovulum, Lewinella, Idiomarina, Pseudidiomarina, Chelatococcus, Aliidiomarina, and Alkalimonas were affected by fewer physicochemical factors.” Does this also mean they had a wide range of adaptability? Or does this mean they weren’t positively correlated with particular nutrients?

- “…have a positive correlation with microbial community structure.” What is a positive correlation with microbial community structure? How do they influence the community structure?

**​​​​​​​Response to conclusion section: **I have rewritten the entire conclusion in a way that captures the conclusive elements of both diversity across seasons and sampling sites and the effects of the environmental factors on the population dynamics of microorganisms in Lake Magadi. Your comments were truly helpful.
